# Isolation of Bioactive Compounds from *Calicotome villosa* Stems

**DOI:** 10.3390/molecules23040851

**Published:** 2018-04-08

**Authors:** Josiane Alhage, Hoda Elbitar, Samir Taha, Jean-Paul Guegan, Zeina Dassouki, Thomas Vives, Thierry Benvegnu

**Affiliations:** 1 AZM Centre for Research in Biotechnology and Its Applications, Laboratory of Applied Biotechnology for Biomolecules, Biotherapy and Bioprocess, Lebanese University, El Mitein Street, Tripoli, Lebanon; josiane.alhage@ensc-rennes.fr (J.A.); samirtaha@ul.edu.lb (S.T.); zeina_dassouki@hotmail.com (Z.D.); 2 ENSCR, CNRS, ISCR (Institut des Sciences Chimiques de Rennes)–UMR 6226, Univ Rennes, 35000 Rennes, France; jean-paul.guegan@ensc-rennes.fr (J.-P.G.); thomas.vives@ensc-rennes.fr (T.V.); 3 Faculty of Public Health, Rafic Hariri Campus, Lebanese University, Hadath, Beyrouth, Lebanon

**Keywords:** *Calicotome villosa*, basalethanoïd B, chrysine-7-*O*-β-d-glucopyranoside, calythropsin, antibacterial, antidiabetic, cytotoxicity, HeLa

## Abstract

A phenylethanoid, two steroids, a flavone glucoside and a chalcone have been isolated for the first time from the stems of *Calicotome villosa* together with a previously isolated flavone glucoside. Their structures were determined by spectroscopic analyses (NMR, HRMS) as basalethanoïd B (**1**), β-sitosterol and stigmasterol (**2**), chrysine-7-*O*-β-d-glucopyranoside (**3**), chrysine 7-((6′′-*O*-acetyl)-*O*-β-d-glucopyranoside) (**4**) and calythropsin (**5**). The crude extracts and the isolated compounds (except 4), were evaluated for their antioxidant, antimicrobial (against two Gram-positive bacterial strains: *Staphylococcus aureus*, *Bacillus cereus*, four Gram-negative bacterial strains: *Staphylococcus epidermidis*, *Klebsiella pneumonia*, *Acinetobacter baumanii*, and three yeasts: *Candida albicans*, *Candida tropicalis*, and *Candida glabrata*), hemolytic, antidiabetic, anti-inflammatory and cytotoxic activity. The crude extracts showed good ability to scavenge the free radical DPPH. Methanol stem extract followed by the dichloromethane stem extract showed moderate antimicrobial potency; furthermore, at 1 mg/mL the methanol extract showed an inhibition of *C. albicans* growth comparable to nystatin. Dichloromethane, methanol, and aqueous extracts inhibited 98%, 90%, and 80% of HeLa cell proliferation at 2 mg/mL respectively. Weak hypoglycemic and hemolytic effects were exhibited by the crude extracts. Among all the tested compounds, compound **3** showed remarkable hypoglycemic potential (93% at 0.1 mg/mL) followed by compound **5** (90% at 0.3 mg/mL). Compound **5** was the most effective in the DPPH**^.^** scavenging assay (100% at 0.1 mg/mL) and cytotoxic assay on HeLa cells (99% and 90% after 24 and 48 h of treatment at 0.1 mg/mL, respectively). No anti-inflammatory effects were displayed by any of the crude extracts or the isolated compounds at any of the tested concentrations.

## 1. Introduction

The genus *Calicotome* belongs to the Fabaceae family. Five species of this genus are distributed in the Mediterranean basin (*Calicotome spinosa* (L.) Link, *C. infesta* (*C. Presl*) Guss., *C. villosa* (Poir.) Link, *C. rigida* (Viv.) Rothm. and *C. fontanesii* Rothm.) [1]. *C. villosa* is a thorny shrub that can reach 2 m in height. It has sharp terminations, trifoliate and oval leaves with yellow and grouped flowers [2]. There exists an intermedia subspecies of *Calicotome villosa*, “*C. villosa* subsp. *Intermedia*”. This subspecies grows in Morocco, Algeria and Spain [3,4] while *C. villosa* is common in the Mediterranean area and is the only species of this genus that grows in Lebanon. It is distributed in many regions such as Beirut, Tripoli, Chekka and Akkar [4,5].

This species has been used in Sicilian folk medicine for the treatment of cutaneous abscesses, furuncles, chilblain and as an antitumor agent [4]. Also, the flower infusion is used by the Palestinian population for the treatment of cardiovascular and nervous system disorders [6]. Previous phytochemical investigations on *C. villosa* leaf methanol extract have led to the isolation and identification of many phenolic compounds, alkaloids, and anthraquinones [2,7]. A biological study has shown antimicrobial activity of the leaf methanolic extract of this species against *S. aureus*, *E. coli*, *B. lentus*, *P. aeruginosa*, *P. rettgeri*, and *M. morganii* [2]. Limited studies have been carried out on *C. villosa*’s biological activities and chemical and biological studies of this plant’s stems have not been reported yet. As part of ongoing research to identify bioactive substances from Lebanese medicinal plants, macerated dichloromethane, methanol and aqueous extracts of *C. villosa* stems have been evaluated for their antioxidant, antimicrobial, hemolytic, antidiabetic, anti-inflammatory and cytotoxic activities. Within this context, the present study describes the isolation and the structural elucidation of a phenylethanoid, two steroids, a flavone glucosides and a chalcone for the first time from stems of this species. These isolated compounds were, furthermore, assayed for all the above in vitro bioactivities. No study has been recorded yet concerning all the tested bioactivities on calythropsin. Basalethanoïd B was evaluated for the first time for its hemolytic, antidiabetic, anti-inflammatory and cytotoxic activities. Also, the hemolytic, anti-inflammatory and cytotoxic activities of chrysine-7-*O*-β-d-glucopyranoside are studied for the first time.

## 2. Results

### 2.1. Nomenclature

*C. villosa* stems were collected and dried at room temperature under shade. The dried stems were macerated separately with dichloromethane, methanol, and water for 48 h. The obtained mixtures were then filtered and evaporated to dryness under reduced pressure. Regarding the nomenclature used in this paper, CV refers to the first initials of the plant species, followed by S (the first initial of stems). This letter is followed by M for maceration (the type of extraction), and after it the solvent used for the extraction (CH_2_Cl_2_, CH_3_OH or H_2_O) is mentioned.

### 2.2. Characterization of Compounds from C. villosa Stems

**Figure 1:**
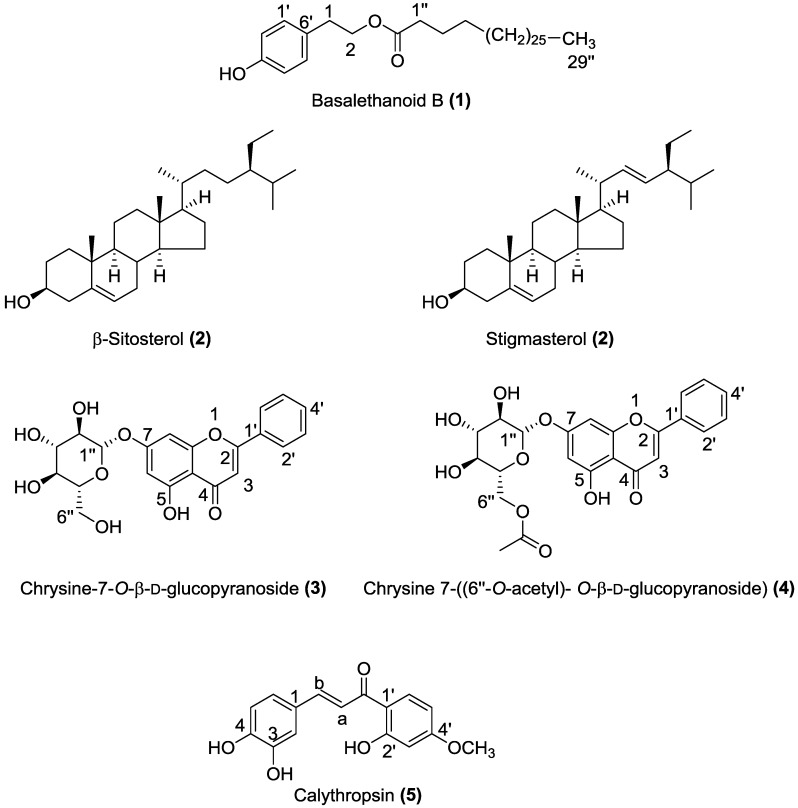
Chemical structures of compounds **1**–**5** isolated from the dichloromethane and methanol extracts of *C. villosa* stems.

The dichloromethane and methanol stem extracts were subjected, separately, to repeated column chromatography on silica gel and preparative normal-phase thin-layer chromatography to yield five compounds (Figure 1)*.*

Compound **1** was isolated as a white amorphous powder. Its molecular formula was determined as C_38_H_68_O_3_ on the basis of HR-ESI-MS (*m*/*z* 595.5 [M + Na]^+^). The compound showed identical NMR signals (Table 1) to those described in the literature by [8]. The ^1^H-NMR spectrum showed the typical AA’XX’ system of a *p*-substituted benzene ring at δ = 7.10 (d, *J* = 8.48 Hz, H-2′, H-6′) and 6.78 (d, *J* = 8.48 Hz, H-3′, H-5′), the presence of a CH_2_CH_2_O unit at δ = 4.25 (t, *J* = 7.04 Hz, H-1), and 2.88 (t, *J* =7.03 Hz, H-2), a terminal methyl at δ = 0.9 (t, *J* = 7.06 Hz), methylenes at δ = 2.29 (t, *J* = 7.4 Hz, -CH_2_-CO-), 1.59 (m, CH_2_-CH_2_-CH_2_-CO-), and 1.27 (br s, nH). These data suggested the presence of a long chain linked to a 4-hydroxyphenylethanol moiety. This inference was supported by the 2D NMR, which showed characteristic signals of a long chain at δ = 173.9 (C=O), 34.2 (C-1′′), 31.9–29.1 (CH_2_)*n*, 28 (C-3′′), and 14 (CH_3_) and of a *p*-disubstituted benzene ring at δ = 115.3 (C-3′, C-5′), 130.0 (C-1′), 130.4 (C-2′, C-6′) and 154.19 (C-4′), and the CH_2_CH_2_O unit at δ = 64.9 (C-1) and 34.3 (C-2) (Table 1) (Figures S1, S2, S3, and S4, Supplementary Materials). From these spectroscopic data compound **1** was characterized as basalethanoïd B.

Compound **2** was isolated as a white powder. HR-ESI-MS showed two [M + Na]^+^ molecular ion peaks at *m*/*z* 437.37 and 435.59, which correspond respectively to C_29_H_50_O and C_29_H_48_O. In ^1^H-NMR, proton H-3 appeared as a multiplet at δ 3.55 ppm and olefinic protons were present at δ 5.37 (m), 5.17 (dd) and 5.04 (dd) (Figure S5, Supplementary Materials). The ^13^C-NMR spectrum revealed characteristic signals of a double bond at 140.81 ppm (C5) and 121.62 ppm (C6). The value at 11.92 ppm corresponds to an angular carbon atom (C28), 138.7 ppm for C-20 and 129.5 ppm for C-21. The alkene carbons appeared at 140.80, 138.35, 129.29 and 121.74 ppm (Figure S6, Supplementary Materials). From all the above spectroscopic data, compound **2** was characterized as a mixture of β-sitosterol and stigmasterol. The only difference between these two compounds was the presence of a double bond between C22 and C23 in stigmasterol. According to literature, it is very difficult to obtain β-sitosterol and stigmasterol in a pure state [9,10,11]. The spectral and physical analyses in the present study (^1^H-NMR, ^13^C-NMR, and HR-ESI-MS) showed a strong similarity to the experimental data available in the literature [11,12,13].

Compound **3** was isolated as a pale yellow powder. Its molecular formula was determined as C_21_ H_20_ O_9_ on the basis of HR-ESI-MS (*m*/*z* 439.10 [M + Na]^+^, 417.11 [M + H]^+^, 255.06 [M − C_6_H_10_O_5_ + H]^+^). It showed identical NMR signals (Table 2) to those described in the literature by [14]. The ^1^H-NMR spectrum exhibited a flavonoid pattern and showed signals at δ 7.06 (1H, s) 6.89 (1H, s) and 6.48 ppm (1H, s) typical of protons at C-3, C-8 and C-6 of a flavone skeleton. Chemical shifts of 8.10 (d, H2′, H6′) and 7.61 (m, H3′, H4′, H5′) suggested that there is no substitution in the B ring of the flavonoid. The signal at δ 12.81 ppm was assigned to the C-5 hydroxyl. ^1^H-NMR shifts at δ 3.19 to 3.73 ppm and signals in the 2D-NMR spectrum just below δ 77 ppm indicated the presence of a glucose moiety. The signal at δ 5.09 ppm was assigned to the anomeric proton (H-1”) with a coupling constant (*J* = 7.37 Hz) indicating a β-configuration [14,15] (Figures S7, S8, and S9, Supplementary Materials). From these data, and according to previous literature [3,14,15,16], compound **3** was characterized as chrysine-7-*O*-β-d-glucopyranoside. 

Compound **4** was isolated as pale yellow solid. Its molecular formula was determined as C_23_H_22_O_10_ on the basis of HR-ESI-MS (*m*/*z* 481.11 [M + Na]^+^). It showed identical NMR signals (Table 3) to those described in the literature by [7]. The ^1^H-NMR spectrum exhibited a flavonoid pattern and showed signals at δ 6.84 (1H, s) 6.85 (1H, s) and 6.56 ppm (1H, s) typical of protons at C-3, C-8 and C-6 of a flavone skeleton. Chemical shifts of 8.04 (d, H2′, H6′) and 7.60 (m, H3′, H4′, H5′) suggested that there is no substitution in the B ring of the flavonoid. The signals at 3.25 to 4.41 ppm correspond to a glycosidic moiety; furthermore, at δ 5.06 ppm an anomeric proton appears as a doublet with a coupling constant of *J* = 7.66 Hz indicating a β-linkage of the sugar unit to the aglycone. The cross peak from H-1” (δ 5.08) to C-7 (δ 164.78) in the HMBC spectrum confirmed that the glycosylation takes place in the 7 position. The signals at 4.48, 4.24 and 3.79 correspond to the acetyl group on position 6”. The ^1^H-COSY and HMBC spectra confirmed the above assignments (Figures S10, S11, and S12, Supplementary Materials). From these data, and according to previous literature [3,7] compound **4** was characterized as chrysine 7-(6′′-*O*-acetyl)-*O*-(β-d-glucopyranoside).

Compound **5** was isolated as a yellow oil. HR-ESI-MS showed a [M − H]^+^ molecular ion peak at *m*/*z* 287.09 corresponding to C_16_H_13_O_5._ This compound showed identical NMR signals (Table 4) to those described in the literature by [17]. The ^1^H-NMR data indicated a chalcone molecule due to the presence of signals referring to two conjugated aromatic system [(d, 6.42 ppm, *J* = 2.15 Hz), (dd, 6.36 ppm, *J* = 8.52, 2.15 Hz) (d, 7.48 ppm, *J* = 8.55 Hz)] ; [(d, 7.01 ppm, *J* = 2.15 Hz), (dd, 6.69 ppm, *J* = 8.38, 2.06 Hz) (d, 6.69 ppm, *J* = 8.38 Hz)]. In addition, signals at 7.27 and 7.4 ppm refer to an alkene possessing E-geometry attached to an unsaturated ketone [(d, 7.27 ppm, *J* = 15.8 Hz), (d, 7.40 ppm, *J* = 15.8 Hz)]. This inference was supported by the 2D-NMR spectrum which showed signals at δ = 190 (C=O), 123 (C-Ha), 142 (C-Hb) corresponding to C_a_ et C_b_ of a chalconoid skeleton (Figures S13, S14, S15, and S16, Supplementary Materials). Thus, the structure of compound **5** was elucidated as calythropsin. 

Subsequently, the antioxidant, antimicrobial hemolytic, antidiabetic, anti-inflammatory and cytotoxic activities of *C. villosa* stem extracts and the isolated compounds **1**, **2**, **3**, and **5** were investigated. Compound **4** was available only in a small quantity, therefore, we could not evaluate its bioactivity. 

### 2.3. DPPH Radical Scavenging of C. villosa Stem Extracts and Compounds

**Figure 2:**
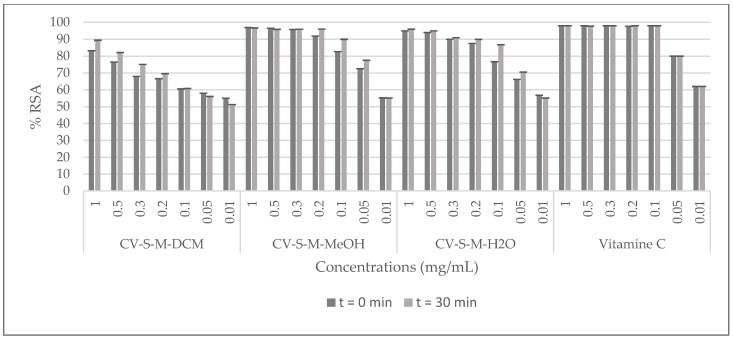
DPPH radical scavenging effects of *C. villosa* stems extracts. All values are expressed as mean of triplicate ± SD.

**Figure 3:**
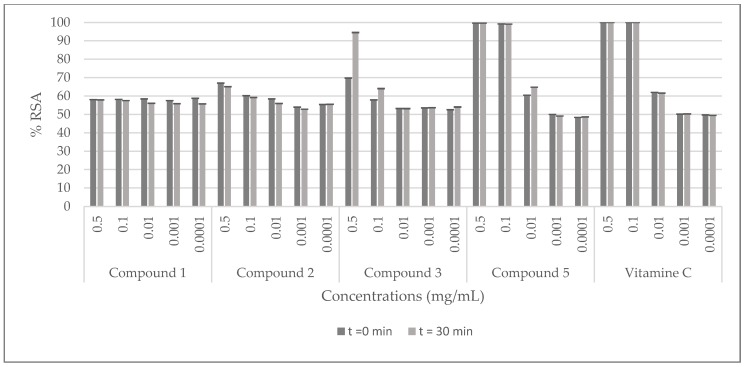
DPPH radical scavenging effects of the compounds isolated from *C. villosa* stems extracts. All values are expressed as mean of triplicate ± SD.

The radical scavenging activity (RSA) of *C. villosa* stem crude extracts and isolated compounds was determined by a 2,2-diphenyl-1-picrylhydrazyl (DPPH) assay, using vitamin C as a standard. The samples were prepared with the solvent of extraction at 0.01, 0.05, 0.1, 0.2, 0.3, 0.5 and 1 mg/mL for the extracts and, at 0.0001, 0.001, 0.01, 0.1, 0.5 mg/mL for the compounds. Then they were added to a methanolic solution of DPPH. The absorbance of the mixture was measured at 0 and 30 min. The antioxidant activity was then calculated by evaluating the decrease in the absorbance. The best activity of the dichloromethane crude extract was shown at 1 mg/mL (inhibition of 90%), while the methanolic and the aqueous extracts showed an inhibition of 90% at 0.2 mg/mL. Furthermore, the obtained scavenging activity of the stem methanol extract was comparable to the positive control at 0.2 mg/mL (Figure 2). Among all the tested compounds, compound **5** (calythropsin) was the most potent molecule and it showed a scavenging activity equivalent to vitamin C at all the concentrations tested (Figure 3).

### 2.4. Antimicrobial Assay

In this study, *C. villosa* stem crude extracts and the isolated compounds were evaluated for their antimicrobial activity by the plate hole diffusion technique against two Gram-positive bacterial strains: *Staphylococcus aureus*, *Bacillus cereus*, four Gram-negative bacterial strains: coagulase-negative Staphylococci from *S. epidermidis*, *Klebsiella pneumonia*, *Acinetobacter baumanii*, and three yeasts: *Candida albicans*, *Candida tropicalis*, and *Candida glabrata.* Extracts were tested at 1, 2, and 10 mg/mL and compounds were tested at 0.001, 0.01, 0.1, and 1 mg/mL. All extracts were active against at least one of the tested strains. Dichloromethane and methanol stem extracts were more active than the aqueous stem extract. At 1 mg/mL, the dichloromethane extract showed 10, 7, 13 and 11 mm inhibition diameters against respectively *B. cereus*, *S. epidermidis*, *C. tropicalis,* and *C. glabrata* growth, while at the same concentration, the methanol extract exhibited 16, 15, 17 and 12 mm inhibition diameters against respectively *K. pneumoniae*, *C. tropicalis*, *C. albicans*, and *C. glabrata* growth. The aqueous extract showed 8 mm inhibition diameter against only *C. albicans.* Furthermore, among all the tested compounds, only compound **3** (chrysine-7-*O*-β-d-glucopyranoside) inhibited the growth of *A. baumanii* at 0.001 mg/mL (10 mm, Table 5). 

### 2.5. Hemolytic Assay

**Figure 4:**
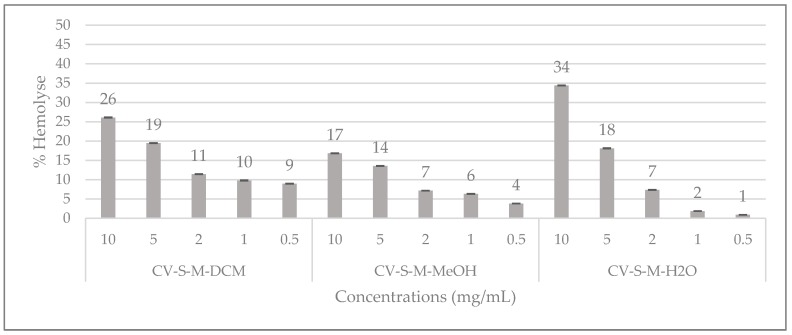
Hemolytic effects of *C. villosa* stems extracts. All values are expressed as mean of triplicate ± SD.

The results of the hemolytic activity of *C. villosa* stem crude extracts are shown in Figure 4. The percentage of hemolysis caused by the extracts at different concentrations is studied and it can be observed that at 10 mg/mL the aqueous extract revealed the highest percent of hemolysis (34%) followed by the dichloromethane extract (26%) and the methanol extract (17%). From 2 mg/mL, the hemolytic percentage is insignificant for all the extracts (Figure 4). The isolated compounds **1**, **2**, **3** and **5** were also evaluated for their hemolytic ability at 0.001, 0.01, 0.1, and 0.5 mg/mL and no hemolytic activity was detected (data not shown).

### 2.6. α-Glucosidase Inhibition Assay

**Figure 5:**
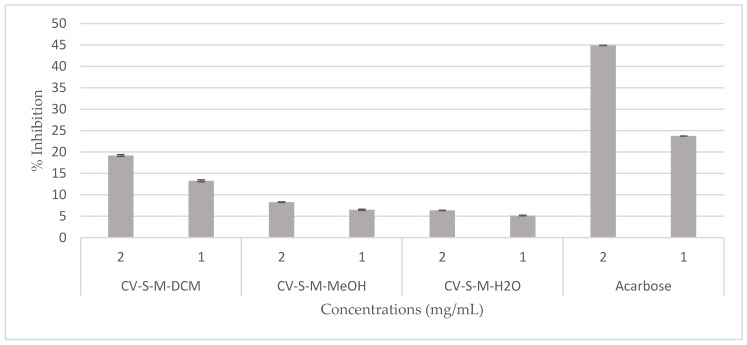
Hypoglycemic effect of the *C. villosa* stems extracts. All values are expressed as mean of triplicate ± SD.

**Figure 6:**
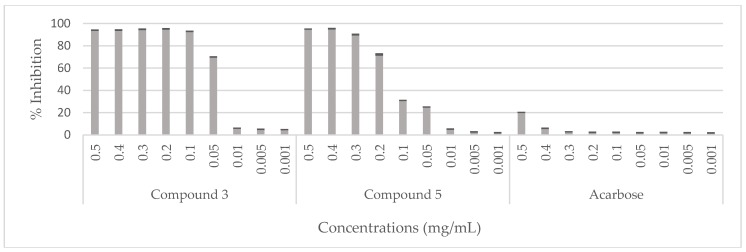
Hypoglycemic effect of compounds (**3** and **5**) isolated from *C. villosa* stems extracts. All values are expressed as mean of triplicate ± SD.

The hypoglycemic effect of the *C. villosa* stem extracts and four isolated compounds **1**, **2**, **3**, and **5** were evaluated using α-glucosidase from *Saccharomyces cerevisiae*. On the one hand, all stem extracts exhibited a weak inhibitory effect while the dichloromethane extract was the most effective extract, showing a 20% inhibition of the enzyme activity (Figure 5). On the other hand, a very strong and remarkable inhibitory effect was revealed by compounds **3** (chrysine-7-*O*-β-d-glucopyranoside) and **5** (calythropsin). These two compounds showed 90% inhibition of the enzyme activity at respectively 0.1 and 0.3 mg/mL. In addition, at 0.05 mg/mL, compound **3** exhibited an inhibition of 70% of the activity of α-glucosidase. Furthermore, the detected activity is much higher than the reference activity. A decrease of the activity was remarked from 0.01 mg/mL for compound **3** and from 0.1 mg/mL for compound **5** (Figure 6). Compounds **1** and **2** displayed no inhibition of the enzyme activity (data not shown).

### 2.7. Anti-Inflammatory Assay

The dichloromethane, methanol and aqueous extracts of *C. villosa* stems and four isolated compounds **1**, **2**, **3**, and **5** were evaluated for their activity against bee venom phospholipase A2. Manoalide was used as a reference standard. The concentrations tested were 1, 2, 5, and 10 mg/mL for the crude extracts and 0.1, 0.3, 0.5, and 1 mg/mL for the isolated compounds. However, all samples were inactive towards all the tested concentrations. 

### 2.8. Cytotoxic Assay

**Figure 7:**
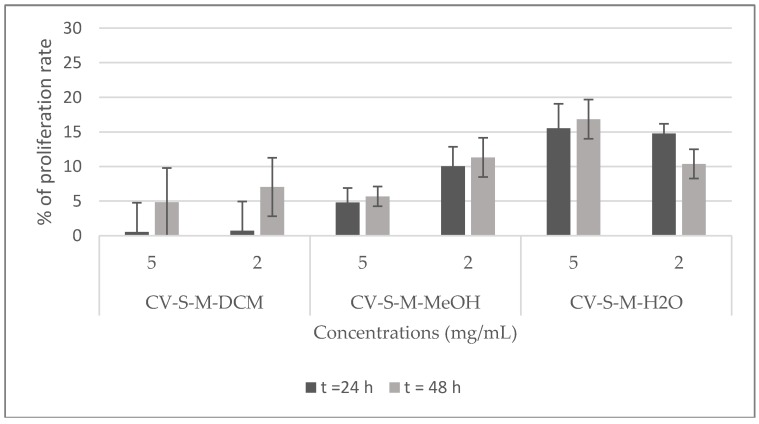
Viability of Hela cells treated with *C. villosa* stems extracts. All values are expressed as mean of duplicate ± SD.

**Figure 8:**
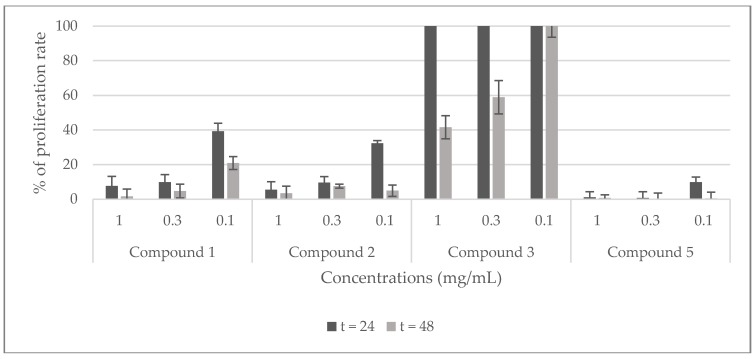
Viability of Hela cells treated with compounds (**1**, **2**, **3**, and **5**) isolated from *C. villosa* stems extracts. All values are expressed as mean of duplicate ± SD.

The potential cytotoxicity of the stem extracts and four isolated compounds **1**, **2**, **3**, and **5** towards Hela cell line was evaluated by the Trypan blue dye exclusion technique. HeLa cells were exposed to different concentrations of the extracts (2 and 5 mg/mL) and of the compounds (0.1, 0.3, and 1 mg/mL) for 24, and 48 h. The percentage of proliferation rate was calculated with respect to untreated cells (100%). The results shown in Figure 7 demonstrate that the viability of Hela cells is strongly inhibited by all the extracts at the two tested concentrations and, it is totally inhibited by the dichloromethane stem extract at 2 mg/mL after 24 h of treatment. Compounds **1** and **2** showed a good inhibition of the proliferation rate (90%) at 0.3, and 1 mg/mL after 24 and 48 h, and at 0.1 mg/mL after 48 h. At the same time, they exhibited a moderate inhibition (<40%, >30%) at 0.1 mg/mL after 24 h. Compound **3** showed no toxicity against this cell line, rather it activates its proliferation. Compound **5** displayed the highest inhibition of the proliferation rate at all the tested concentrations after 24 and 48 h (Figure 8).

## 3. Discussion

The study of medicinal plants represents a universal strategy for the discovery of new therapeutic agents since plants contain an inestimable number of biologically active molecules [18]. Medicinal plants are widely used in traditional medicine for their pharmacological and healing properties. However, an inappropriate use of natural products may be, in some cases, very dangerous; therefore, it is important to associate scientific studies with clinical observations and traditional knowledge in order to set up an inventory of their biological activities. The current work is part of this scenario and its purpose is to screen the biological effectiveness of crude extracts from *C. villosa* stems as well as of four isolated compounds.

No study of the stems of this species was reported to date, as previous evaluations have been carried out on extracts from its leaves and flowers. For that reason, we were interested in investigating this specific part of the plant. The stems of this species were extracted successively by dichloromethane, methanol, and water. Then, the dichloromethane and methanol extracts were subjected to successive purifications on silica gel column chromatography and preparative-TLC to yield five compounds.

The evaluation of different bioactivities of *C. villosa* crude extracts showed that the dichloromethane extract possessed a high scavenging capacity of the radical DPPH**^·^** at 1 mg/mL, while the methanol extract revealed an efficacy comparable to the standard reference at 0.2 mg/mL. However, this extract was slightly more potent than the aqueous one at all the concentrations tested. These results can be explained by the fact that methanol facilitates the extraction of flavonoids widely known for their antioxidant capacity [19]. In addition, they revealed an antimicrobial effect against at least one of the tested strains. Though the aqueous extract showed the lowest efficiency, the dichloromethane and methanol extracts were more potent. This may result from the fact that most compounds that have been found active against antimicrobial strains are not water soluble [20,21]. A remarkable growth inhibition was obtained by the methanol extract towards *C. albicans* with an inhibition zone at 1 mg/mL comparable with that of the Nystatin reference. Further, hemolytic effects of these extracts were significant only at 10 mg/mL. However, no antidiabetic or anti-inflammatory effects were detected by these extracts. Moreover, they inhibited 90% of the HeLa cell proliferation rate at 24 h and 48 h in a somewhat dose-dependent manner. The dichloromethane extract was the most cytotoxic extract, totally inhibiting the proliferation rate at 5 mg/mL after 24 h of treatment. Previous studies reported that the leaf methanol extract of this species possessed antioxidant, antibacterial, antifungal and cytotoxic (on Vero cells) properties [2,22].

Basalethanoïd B (**1**) is a phenylethanoid previously isolated from several species such as *Jacaranda filicifolia* [23], *Fraxinus chinensis Roxb* [24], *Newbouldia laevis* [25,26,27,28], *Phlomis medicinilas* [29], and *Basalmocitrus cameroonensis* [8]. However, it should be noted that it is the first time a phenylethanoid molecule is characterized from the *Calicotome* genus. Few studies were carried out on the biological activities of this compound. Among them, one showed that the basalethanoïd B can inhibit, in vitro, 75% of the acetylcholinesterase activity at 100 μg/mL [30]. Also, it has an immunomodulatory potential comparable to ibuprofen [8]. In this study, the antioxidant, hemolytic, antidiabetic, anti-inflammatory and cytotoxic activities of this compound were evaluated for the first time. The results showed that basalethanoïd B possesses a weak scavenging capacity of the radical DPPH, and the absence of any antidiabetic and anti-inflammatory potency. This latter result is in good agreement with a previous study in which this molecule slightly inhibited the 5-lipoxygenase enzyme that indirectly means it possesses very weak anti-inflammatory potency. On the contrary, basalethanoïd B showed a very good inhibition of HeLa cell proliferation rate at 0.3 mg/mL after 24 and 48 h of treatment. Moreover, our study revealed the absence of any antibacterial and antifungal activity, at all the concentrations tested. Our results are not in accordance with another study that showed a moderate activity of this molecule against the same strains such as *S. aureus*, *B. cereus*, *C. albicans*, and *C. glabrata* [26].

Compound **2** was a mixture of β-sitosterol and stigmasterol. These two molecules are isolated for the first time from this species but they have been already isolated from many species such as *Parkia speciosa* [31], *Heliotropium ellipticum* [32], *Heliotropium marifolium* [33], *Calotropis gigantea* [13], *Ageratum conyzoides* [9], and *Acacia nilotica* [12]. Previous biological evaluations validated their antioxidant, antibacterial, anti-inflammatory and cytotoxic effects [34,35,36]. In our hands, the mixture of these two compounds showed weak antioxidant potency. Furthermore, they exhibited no antibacterial, hemolytic or anti-inflammatory effects. However, they displayed moderate cytotoxic activity towards HeLa cells. 

Chrysine-7-*O*-β-d-glucopyranoside (**3**) is a flavone glucoside previously isolated from *Populous deltoids* [37], *Scutellaria orientalis* [38], *Enkianthus subsessilis* [39], *Sarothamnus patens* [40], *Spartium junceum* [16], *Cherry stalk* [41] and from many other species. It was also isolated from *C. villosa* subsp. *Intermedia* [3] but this is the first time it has been isolated from this species. Previous studies showed hypotensive and diuretic properties of this compound and they revealed an ability to increase the α-transcriptional activity in MCF-7 cells [42,43]. In our work, a weak antioxidant activity toward the radical DPPH^.^ was observed; these results are in agreement with a previous study that showed similar results [44]. In addition, it inhibited by 10 mm the growth of *A. baumanii* at 0.001 mg/mL. This result is in contrast with the fact that flavone glucosides usually possess weak antimicrobial potency [14]. In addition, it inhibited 90% and 70% of α-glucosidase activity at respectively 0.1 and 0.05 mg/mL. A decrease of the activity was observed from 0.01 and 0.1 mg/mL for compound **3** and compound **5 **respectively Nevertheless, it was much more efficient than the standard reference at all the concentrations tested. Consequently, compound **3** appears as a promising hypoglycemic agent. Many reports support our findings concerning the low inhibition exhibited by acarbose, where it also showed poor inhibitory effect against baker’s yeast [45,46]. However, it did not display any hemolytic or anti-inflammatory effect. On the other hand, it was the only compound tested, that instead of inhibiting the proliferation rate of Hela cells, it activated their division. This can be justified by the presence of the glucose moiety in its structure; previous studies demonstrated that the increasing number of sugar moieties in a compound structure reduces its cytotoxic potency [47,48].

Calythropsin (**5**) is a chalcone isolated for the first time from the *Calicotome* genus. It was previously isolated from three other species (*Calythropsis aurea* [17], *Faramea salicifolia* [49], *Bauhinia glauca* subsp. *Pernervosa* [50]. It is frequently produced in biosynthetic pathways. Actually, the biological properties and the easy synthetic routes of chalcones have attracted the interest of researchers. Previous reviews described the significant pharmacological potentials of chalcones derivatives. The efficacy of these compounds strongly depends on the variation of the functions present in the structure. However, the synthetic derivatives of calythropsin exhibited more cytotoxicity than the calythropsin itself [17,51,52,53,54,55]. 

Among all the tested compounds, only calythropsin showed a radical scavenging capacity comparable to the reference at all the concentrations tested. This may be explained by the previously reported free radicals scavenging property of chalcone molecules [56,57]. No antimicrobial, hemolytic, or anti-inflammatory activities were detected for this compound. However, it showed the most cytotoxic effect against HeLa cells where it totally inhibited the proliferation of the cells at 0.3 mg/mL after 24 and 48 h of treatment and at 0.1 mg/mL after 48 h. 

Other interpretations can be made by focusing on the initial activities obtained from the crude extracts and the final ones displayed by the isolated compounds. As we can observe, in some cases isolated compounds exhibit higher potentials than the crude extracts such like chrysine-7-*O*-β-d-glucopyranoside and calythropsin that exhibited remarkable inhibitions of the α-glucosidase enzyme while the methanol extract showed negligible inhibition. This may be due to the low quantities of these two compounds present in the initial sample or because of the presence in the extract of additional compounds having antagonist effects. Also, we can see similar results in the cytotoxic assay, where chrysine-7-*O*-β-d-glucopyranoside showed an activation of the proliferation rate of Hela cells but the crude extract showed a potential inhibition of the proliferation. On the contrary, some initial activities of the extracts were not found in the isolated compounds as for the dichloromethane extract and its isolated compounds (basalethanoïd B, β-sitosterol and stigmasterol) where the ability to scavenge the free radical (DPPH^.^) remarkably decreased. This can be explained by the fact that natural substances present in a crude extract act together in a synergystic effect. 

## 4. Materials and Methods 

### 4.1. General Information

2,2-Diphenyl-1-picrylhydrazyl (DPPH) (97%), vitamin C (99%), α-glucosidase from *S. cerevisiae* (10.8 U/mg), *p*-nitrophenyl α-d-glucopyranoside (>99%), acarbose (>95%) and PLA2 from *Apis mellifera* (600–2400 U/mg), were purchased from Sigma-Aldrich (Saint-Quentin-Fallavier, France). Manoalide (>98%) was purchased from Abcam (Cambridge, MA, USA). NMR spectra were obtained in chloroform-d1, dichloromethane-d2, methanol-d4 or DMSOd6 at 298 K on an Avance III 400 spectrometer (Bruker, Wasserbourg, France) operating at 400.13 MHz for ^1^H, equipped with a BBFO probe with a Z-gradient coil and a GREAT 1/10 gradient unit. Chemical shifts are given in δ units (ppm) measured downfield from Me_4_Si as external reference. The HRMS was measured at the CRMPO (University of Rennes 1, France). Silica gel (UNI-Chem Si 60 (200–300 mesh)) was used for column chromatography. Analytical thin-layer chromatography (TLC) was carried out on 60 F254 silica gel non-activated plates (E. Merck, Lyon, France). All procedures were carried out at room temperature using solvents purchased from reliable commercial sources and employed without further purification.

### 4.2. Plant Material

The whole plant of *C. villosa* was collected in Akkar, North of Lebanon, in February 2016 and identified by Professors Georges and Henriette Tohme (professors in natural substances). Plant identification was based on plant morphology and habitat ecology. The stems of this species were dried in the shade at room temperature for two weeks and stored in a dry place.

### 4.3. Extraction and Isolation

The air-dried stems of *C. villosa* were macerated for 48 h at room temperature by successively dichloromethane, methanol, and water. The different extracts were filtered and concentrated under reduced pressure to dryness. 

The stem dichloromethane extract (9 g) was subjected to a silica gel column chromatography using a cyclohexane-CH_2_Cl_2_ gradient system (100:0–0:100) followed by a CH_2_Cl_2_-MeOH gradient system (100:0–20:80). A total of 356 tubes were collected (50 mL each) and analysed with silica gel F254 TLC plates using cyclohexane/CH_2_Cl_2_ and CH_2_Cl_2_/MeOH as the developing solvent systems. Compounds were visualized under UV light at the wavelength of 254 and 365 nm.

Chromatograms were sprayed with vanillin sulphuric solution and heated over a hotplate. Similar tubes were pooled together to yield 15 major fractions (Fractions 1–15). F_9_ (652 mg), which was eluted with cyclohexane/CH_2_Cl_2_ (30:70), was then subjected to a purification using a successive washing by pentane and dichloromethane. The precipitate of this fraction yield compound **1** (150 mg). The pentane soluble fraction (F_9-pentane_, 100 mg) was then subjected to a purification by preparative normal-phase thin-layer chromatography with CH_2_Cl_2_-MeOH solvent system (98:2) as mobile phase to obtain compound **2** (26 mg). 

The stem methanol extract (40 g) was subjected to a silica gel column chromatography using Cyclohexane-CH_2_Cl_2_ gradient system (100:0–0:100) followed by a CH_2_Cl_2_-MeOH gradient system (100:0–0:100) followed by a MeOH-H_2_O system (100:0–90:10) to give 712 tubes (50 mL each). According to the TLC profiles, similar tubes were assembled and 15 fractions came out (Fractions 1–15). Fractions 9 and 10, which were eluted with CH_2_Cl_2_/MeOH (90:10), were combined (6.7 g) and subjected to a purification using a successive washing by MeOH and H_2_O. The precipitate yield compound **3** (500 mg). The MeOH soluble fraction (F_9.10-MeOH_, 6 g) was then subjected to a normal-phase silica gel CC using a cyclohexane-EtOAc solvent system (100:0–0:100), followed by EtOAc-MeOH solvent system (100:0–50:50) to give 18 fractions (Fractions A–R). Fractions L to P (F_L→P_, 240 mg) were then subjected to a purification by preparative normal-phase thin-layer chromatography with cyclohexane-EtOAc solvent system (10:90) as mobile phase to obtain compound **4** (2.8 mg) and compound **5** (16 mg). The spectroscopic data of the isolated compounds were as follows: 

*Basalethanoïd B* (**1**). White amorphous powder. ^1^H-NMR and ^13^C-NMR (400 MHz, CDCl_3_) see Table 1. ESI-MS *m*/*z*: 595.50 [M + Na]^+^, 611.48 [M + K]^+^ (calculated for C_38_H_68_O_3_Na, 595.50).

*β-Sitosterol and Stigmasterol* (**2**): white powder. ^1^H-NMR (400 MHz, CDCl_3_) has given signals at δ3.55 (1H, m, H-3), δ5.37 (1H, m, H-6), δ 5.17 (1H, dd, H-23), 5.04 (1H, dd, H-22), 2.37 (1H, m, H-20), δ1.8–2.0 (5H, m) ppm. Other peaks were also observed at δ 0.76–1.42. and ^13^C-NMR (400 MHz, CDCl_3_) has given signals at 140.81 (C-5), 138.35 (C-22), 121.62 (C-6), 129.29 (C-21), 71.83 (C-3), 56.7 (C-14), 56.04 (C-17), 51.24 (C-9), 50.13 (C-9), 40.49 (C-20), 39.78 (C-12), 39.69 (C-13), 37.26 (C-4). ESI-MS: β-sitosterol *m*/*z*: 437.37 [M + Na]^+^ (calculated for C_29_H_50_ONa, 437.3754), stigmasterol *m*/*z*: 435.35 [M + Na]^+^ (calculated for C_29_H_48_ONa, 435.35).

*Chrysine-7-O-β-d-glucopyranoside* (**3**): yellow amorphous powder. ^1^H-NMR and ^13^C-NMR (400 MHz, DMSO) see Table 2. ESI-MS *m*/*z*: 439.10 [M + Na]^+^, 417.11 [M + H]^+^, 255.06 [M-C_6_H_10_O_5_ + H]^+^) (calculated for C_21_H_20_O_9_Na, 439.10).

*Chrysine 7-(6′′-O-acetyl)-O-(β-d-glucopyranoside)* (**4**): pale yellow powder. ^1^H-NMR and ^13^C-NMR (400 MHz, MeOD) see Table 3. ESI-MS *m*/*z*: 481.11 [M + Na]^+^ (calculated for C_23_H_22_O_10_Na, 481.11). 

*Calythropsin* (**5**): yellow oil. ^1^H-NMR and ^13^C-NMR (400 MHz, MeOD) see Table 4. ESI-MS *m*/*z*: 287.09 [M + Na]^+^, 287.09 [M + H]^+^ (calculated for C_16_H_14_O_5_Na, 309.07).

### 4.4. DPPH Radical Scavenging Assay

The hydrogen-donating ability of *C. villosa* stem extracts and compounds **1**, **2**, **3** and **5** was evaluated using the free radical 2,2-diphenyl-1-picrylhydrazyl (DPPH^.^) according to the method described in the literature by Chandini [58,59]. Different concentrations of each sample (0.01, 0.05, 0.1, 0.2, 0.3, 0.5 and 1 mg/mL) and each compound (0.0001, 0.001, 0.01, 0.1 and 0.5) were prepared in the solvent of extraction. 1 mL of 0.16 mM DPPH solution was mixed with 1 mL of the sample solutions. The mixture was incubated at 37 °C for 30 min in the dark, then the absorbance was read at 517 nm. The efficacy to scavenge the DPPH radical was calculated using the following equation:

(1) $$\%\;\mathrm{Inhibition}=\frac{\left(A\;\mathrm{control}-A\;\mathrm{sample}\right)}{A\;\mathrm{control}}\times 100.$$

where A control is the absorbance of the control (DPPH solution without sample), and A sample is the absorbance of the test sample (DPPH solution with test sample) at 517 nm. Triplicate measurements were carried out. Vitamin C was used as positive control.

### 4.5. Antimicrobial Assay

The inhibition zones were determined by hole diffusion using a cell suspension of about 1.5 × 10^6^ CFU/mL obtained from a McFarland turbidity standard N° 0.5. The suspension was standardized by adjusting the optical density to 0.1 at 600 nm (UV-120-01 spectrophotometer, Shimadzu, Kyoto, Japan) [60]. A hole of 6 mm diameter was then made on the plate (8 mm thick). 50 μL of the different extracts (1, 2, and 10 mg/mL) and compounds (0.001, 0.01, 0.1, and 1 mg/mL) solubilized in DMSO was added to each well in the specific position. The negative control (DMSO), and the positive control (antibiotic or antifungal depending on the test) were added in two different wells. The inoculated plates were incubated at 37 °C for 24 h. Antimicrobial activity was evaluated by measuring the diameter of the zone of growth inhibition around the hole. The assay was repeated twice.

Tests were performed in Mueller–Hinton agar against five bacterial reference strains: Gram-positive bacteria: *Staphylococcus aureus*, *Bacillus cereus*, Gram-negative bacteria: *Staphylococcus epidermidis*, *Klebsiella pneumonia*, *Acinetobacter baumanii*, and in Sabouraud agar against three yeasts: *Candida albicans*, *Candida tropicalis*, and *Candida glabrata.* The microorganisms were obtained from the Health and Environment Microbiology laboratory at the AZM Center for Research on Biotechnology Sciences and its Applications (Tripoli, Lebanon).

### 4.6. Hemolytic Assay

The hemolytic activity of *C. villosa* stem extracts on red blood cells was evaluated as described by [61] using fresh human blood. Blood was collected aseptically from healthy volunteers into tubes treated with EDTA and centrifuged at 3000 rpm for 5 min. The obtained supernatant was removed in order to get rid of the plasma and all the components of the blood except the red blood cells. Then pellets are washed with PBS by repeated centrifugation at 3000 RPM for 5 min. Different concentrations of the extracts (0.5, 1, 2, 5, and 10 mg/mL) and the compounds (0.0001, 0.001, 0.01, 0.1, and 0.5, mg/mL) were prepared in the solvent of extraction. Three hundred μL of thirty percent of red blood cells suspension (prepared in PBS) is added to an Ependorff tube containing a disc imbibed with the sample. The tube considered as positive control contains 200 μL of distilled water and 100 μL of red blood cells suspension, whereas the negative control contains 200 μL of PBS and 100 μL of red blood cells. All tubes were then incubated at 4 °C for 60 min. The volume of reaction mixture was made up to 1 mL by adding 700 μL of PBS and centrifuged at 3000 rpm for 5 min. Absorbance of the supernatants was measured at 540 nm and the values obtained with the positive control represented a hemolysis of 100%. Triplicate measurements were carried out. The percentage of hemolysis was then calculated using the following formula:

(2) $$\%\mathrm{Hemolysis}=\left[\frac{\mathrm{Atube}-A\;\mathrm{negative}\;\mathrm{control}\;}{A\;\mathrm{positive}\;\mathrm{control}}\right]\times 100\;$$

### 4.7. α-glucosidase Inhibition Assay

The inhibitory activity of α-glucosidase was determined by slight modifications of the procedure previously reported [62]. Shortly, 15 μL of the enzyme (0.5 U/mL in 0.1 M phosphate buffer (pH-6.8)) and 15 μL of the samples were successively added to 96-well plates and were incubated together at 37 °C for 10 min. Then, 15 μL of the substrate *p-*nitrophenyl α-d-glucopyranoside (*p*-PNP-G) (0.5 mM solution in 0.1 M phosphate buffer (pH-6.8)) was added and the enzymatic reaction was performed at 37 °C for 30 min, after that 100 μL of sodium carbonate (0.2 M) was added as a stop buffer. Finally, the solvent was monitored spectrophotometrically by measuring the absorbance at 405 nm. Acarbose was used as a positive control and the uninhibited enzyme was taken as a negative control. The assay was performed in triplicate. The extracts and fractions were dissolved in DMSO and diluted to desired concentrations with 0.1 M phosphate buffer. The final DMSO concentration was maintained below 2% (*v*/*v*), which was found not to have any effect on the enzyme activities. Different concentrations were tested of each crude extract (0.1, 0.2, 0.3, 0.4, 0.5, 1 and 2 mg/mL) and compounds (0.001, 0.005, 0.01, 0.05, 0.1, 0.2, 0.3, 0.4, 0.5 mg/mL). Appropriate blank was used for all the extracts. 

The α-glucosidase inhibition percentage was calculated as follows:

(3) $$\%\;\mathrm{Inhibition}=\frac{\mathrm{Absorbance}\;\mathrm{of}\;\mathrm{control}-\mathrm{Absorbance}\;\mathrm{of}\;\mathrm{sample}}{\mathrm{Absorbance}\;\mathrm{of}\;\mathrm{control}}\times 100.$$

### 4.8. Anti-Inflammatory Assay

The inhibitory activity of PLA2 (from *Apis mellifera* venom, Sigma-Aldrich) was determined colorimetrically in 96 well plates according to the method described by De Aranjo and Radvany [63] with slight modifications. Briefly, the substrate consisted of 3.5 mM L-α-phosphatidylcholine, 10 mM CaCl_2_, 100 mM NaCl, 7 mM Triton-X100 (Triton, Frankfurt, Germany) and 0.055 mM red phenol as colorimetric indicator. The pH of the reaction mixture was adjusted to 7.6 with 40 mM NaOH solution in a final volume of 100 mL. 10 μL of different concentrations of the extracts (1, 2, 5, and 10 mg/mL) and the compounds (0.1, 0.3, 0.5, and 1 mg/mL) was incubated with 1 μL of the enzymatic solution (1 mg/mL) for 1 h at 37 °C. Then 200 μL of the substrate solution was added. The mixture was incubated for 5 min at 37 °C. Triplicate measurements were carried out. Manoalide was used as a positive control. Kinetic hydrolysis was performed for 5 min, and optical density was read at 558 nm. The PLA2 inhibitory activity was expressed in inhibition percentage and was calculated as follows:

(4) $$\%\;\mathrm{Inhibition}=\left(1-\frac{\left(A\;\left(P0\right)-A\;\left(P5\right)\right)}{\left(A\;\left(T0\right)-A\;\left(T5\right)\right)}\right)\times 100.$$

where A(P0) is the absorbance of the product at t = 0 min, A(P5) is the absorbance of the product at t = 5 min, A(T0) is the absorbance of the control experiment (DMSO without test sample) at t = 0 min and A(T5) is the absorbance of the control experiment (DMSO without test sample) at t = 5 min at 558 nm. 

### 4.9. Cytotoxic Assay

Cytotoxic assay towards a HeLa cell line provided by the American University of Beirut, was carried out according to a previously described method [64]. Hela log phase cells were seeded in a 96-well plate at a concentration of 10^4^ cells/100 μL and incubated at 37 °C for 24 h before the addition of 100 μL of the sample diluted with Dulbecco modified Eagle medium‎, at the desired concentrations for extracts (2, and 5 mg/mL) and compounds (0.1, 0.3, and 1 mg/mL). Cell viability was evaluated after 24 and 48 h, and the final DMSO concentration did not exceed 1%. HeLa cells were grown in Dulbecco modified eagle medium supplemented with 10% foetal bovine serum, 2 mM glutamine, and antibiotics. The Trypan blue dye exclusion test was performed by adding one part of a Trypan blue solution (0.4%) to one part of cell suspension. The percentage of stained cells was determined using a Malassez counting chamber. Cells growth control was performed using the DMEM alone or with DMSO instead of plant extract. All experiments were performed in duplicate. The percentage of proliferation rate was then calculated with respect to not treated cells (100%) using the following formula:

(5) $$\%\;\mathrm{of}\;\mathrm{proliferation}=100\times \frac{\frac{\mathrm{Living}\;\mathrm{cells}}{\mathrm{Dead}\;\mathrm{cells}}\;\mathrm{of}\;\mathrm{treated}\;\mathrm{sample}}{\frac{\mathrm{Living}\;\mathrm{cells}}{\mathrm{Dead}\;\mathrm{cells}}\;\mathrm{of}\;\mathrm{untreated}\;\mathrm{control}}$$

## 5. Conclusions

In conclusion, two compounds isolated from the dichloromethane stem extract of *C. villosa* and two isolated from its methanol stem extract, were characterized and evaluated, together with the crude extracts, for their in vitro biological activities. The crude extracts showed good ability to scavenge the free radical DPPH meanwhile, compound **5** was the most effective with a 100% scavenging activity at 0.1 mg/mL. Also, dichloromethane and methanol crude extracts showed better antimicrobial effects than the aqueous one; furthermore, the methanol extract showed at 1 mg/mL a growth inhibition of *C. albicans* comparatively relative to nystatin. However, among all the tested compounds, compound **3** was the only one to show an inhibition of the growth of *A. baumanii* at 0.001 mg/mL (10 mm). In addition, no remarkable hemolytic or anti-inflammatory activities were detected by the crude extracts or the compounds. Crude extracts showed weak antidiabetic effect while compound **3** showed high potential in inhibiting the activity of α-glucosidase (93% at 0.1 mg/mL) followed by compound **5** (90% at 0.3 mg/mL). Dichloromethane stem extract was the most effective extract toward cytotoxicity on HeLa cells, it showed a total inhibition at 5 mg/mL and a 90% inhibition at 2 mg/mL. Methanol and aqueous extracts inhibited respectively 90% and 80% of HeLa cells proliferation. Compound **5** showed a high toxicity on Hela cells at all the tested concentrations while compound **3** was the only sample to activate the proliferation of this cell line. Finally, this is the first report to assess in vitro biological effects of *C. villosa* stem extracts and of four isolated compounds. However further studies are required to purify other compounds from dichloromethane and methanol crude extracts that may be responsible for the displayed initial properties. Also, chrysin-7-*O*-β-d-glucopyranoside should be subjected to future studies on the mammalian α-glucosidase enzyme to validate its hypoglycemic effect.

## Figures and Tables

**Table 1:** ^1^H-NMR and ^13^C-NMR data for basalethanoïd B (**1**) [8].

	δ_H_ (*J* in Hz)		δ_C_	
Position	Literature * (CDCl_3_)	Experimental ** (CDCl_3_)	Literature * (CDCl_3_)	Experimental ** (CDCl_3_)
1	4.22, t, (6.9)	4.25, t, (7.04)	65.0	64.9
2	2.84, t, (6.9)	2.88, t, (7.03)	34.2	34.3
1′	-	-	128.2	130.0
2′	7.04, d, (8.1)	7.10, d, (8.48)	129.7	130.4
3′	6.79, d, (8.1)	6.78, d, (8.48)	115.4	115.3
4′	-	-	155.7	154.19
5′	6.79, d, (8.1)	6.78, d, (8.48)	115.4	115.3
6′	7.04, d, (8.1)	7.10, d, (8.48)	129.7	130.4
HO-4′	8.53 (s)	-	-	-
1′′-CO	-	*-*	173.7	173.9
1′′	2.29, t,(7.5)	2.29, t, (7.4)	-	34.2
2′′	1.58 (m)	1.59 (m)	34.1	-
3′′			24.8	-
4′′ → 28′′	1.25 (s)	1.27 (s)	31.8–29.3	31.9–29.1
29′′	0.89, t, (6.9)	0.90, t, (7.06)	14.0	14.0

* 500 MHz/125 MHz, ** 400 MHz/100 MHz

**Table 2:** ^1^H-NMR and ^13^C-NMR data of chrysine-7-*O*-β-d-glucopyranoside (**3**) [14].

	δ_H_ (*J* in Hz)		δ_C_	
Position	Literature * (DMSO)	Experimental ** (DMSO)	Literature * (DMSO)	Experimental ** (DMSO)
1′	-	-	130.7	131.06
2′	8.09, d, (7.0)	8.10, d, (6.88)	126.5	127.01
3′	7.59 (m)	7.61 (m)	129.1	129.69
4′			132.1	132.75
5′			129.2	129.69
6′	8.09, d, (7.0)	8.10, d, (6.88)	126.3	127.01
2	-	-	-	-
3	6.95 (s)	7.06 (s)	105.4	106.07
4	-	-	-	182.66
5	-	-	99.5	100.19
5-OH	12.81 (s)	12.81 (s)	-	-
6	6.39 (s)	6.48 (s)	-	-
7	-	-	163.9	163.67
8	6.82 (s)	6.89 (s)	94.7	95.47
1′′	5.12, d, (7.0)	5.09, d, (7.37)	99.7	100.33
2′′	3.19–3.70	3.19-3.72	72.9	76.85
3′′			76.6	73.57
4′′			69.2	70.08
5′′	-	-	-	-
6′′	-	-	-	-
2′′-OH	5.44, d, (4.6)	5.44	72.9	76.85
3′′-OH	5.17, d, (4.2)	5.16	76.6	73.65
4′′-OH	5.10, d, (5.0)	-	69.2	77.5
5′′-H	-	3.48	77.4	77.63
6′′-H	-	3,71		
6′′-OH	4.64, t, (5.5)	4.64	60.7	61.07

* 500 MHz/125 MHz, ** 400 MHz/100 MHz

**Table 3:** ^1^H-NMR and ^13^C-NMR data of chrysine 7-(6′′-*O*-acetyl)-*O*-(β-d-glucopyranoside) (**4**) [7].

	δ_H_ (*J* in Hz)		δ_C_	
Position	Literature * (DMSO)	Experimental ** (CD_3_OD)	Literature * (DMSO)	Experimental ** (CD_3_OD)
1′	-	-	-	-
2′	8.06, d, (7.3)	8.04, d, (8.19)	126.5	126.17
3′	7.58, m	7.60, m	-	128.89
4′			129.2	131.93
5′			132.2	128.89
6′	8.06, d, (7.3)	8.04, d, (8.19)	129.2	126.17
2	-	-	162.9	163.19
3	7.02, s	6.84, s	105.5	105.07
4	-	-	182.2	182.91
5	-	-	161.1	-
6	6.45, bs	6.56, d, (2.17)	99.4	99.86
7	-	-	163.7	164.78
8	6.84, bs	6.85, d, (2.18)	95.0	94.77
9	-	-	157.1	157.2
1′′	4.98, d, (7.4)	5.08, d, (7.66)	99.6	99.98
2′′	-	3.52, m	73.2	73.4
3′′	-		76.3	76.2
4′′	-	3.40, m	69.8	70.3
5′′	-	3.79, dtd	73.9	73.9
6′′	-	4.48,dd	63.4	63.6
	-	4.24, dd		
6′′-CO-CH_3_	-	-	170.2	171.19
6′′-CO-CH_3_	2.02, s	2.08, s	20.1	19.3

* 200 MHz/200 MHz, ** 400 MHz/100 MHz

**Table 4:** ^1^H-NMR and ^13^C-NMR data for calythropsin (**5**) [17].

	δ_H_ (*J* in Hz)		δ_C_	
Position	Literature * (DMSO)	Experimental * (CD_3_OD)	Literature * (DMSO)	Experimental * (CD_3_OD)
C=O	-	-	188.66	191.74
a	7.28, d (15.8)	7.36, d, (15.66)	123.73	123.67
b	7.37, d, (15.8)	7.49, d, (15.66)	141.72	143.20
1	-	-	126.44	121.88
2	7.07, d, (2.0)	7.10, d, (2.10)	114.38	113.84
3	-	-	145.58	144.25
4	-	-	148.20	148.99
5	6.76, d, (8.2)	6.79, d, (8.05)	115.82	115.17
6	6.97, dd, (8.2, 2.0)	6.99, dd, (8.34, 2.09)	121.63	121.96
1′	-	-	120.22	120.35
2′	-	-	162.55	163.07
3′	6.49, d, (1.9)	6.52, d, (2.17)	99.25	98.85
4′	-	-	160.37	161.01
5′	6.44, dd, (8.3, 1.9)	6.45, dd, (8.62, 2.17)	107.88	107.55
6′	7.5, d, (8.3)	7.57, d, (8.56)	132.23	132.32
4′-OMe	3.83 (s)	3.90	55.65	54.72

* 500 MHz/125 MHz,** 400 MHz/100 MHz

**Table 5:** Antimicrobial effects of *C. villosa* stems crude extracts, the isolated compounds, the antibiotics and the antifungal (diameter of the well is 6 mm). All values are expressed as mean of duplicate ± SD.

Nystatin	Ceftazidime	Vancomycine	C5	C3	C2	C1	CV-S-M-H_2_O			CV-S-M-MeOH			CV-S-M-DCM			Strains
Inhibition Diameter Zone (mm)/Concentration (mg/mL)																
0.033	0.2	0.6	0.001				1	2	10	1	2	10	1	2	10	
-	-	20 ± 2.8	-	-	-	-	0	0	0	0	0	11 ± 2.8	10 ± 1.4	11 ± 1.4	13 ± 0.7	*Bacillus cereus*
-	-	21 ± 0.7	-	-	-	-	0	0	0	0	0	8 ± 1.4	7 ± 0.7	9 ± 0.7	0	*Staphylococcus epidermidis*
-	-	18 ± 1.4	-	-	-	-	0	0	0	0	0	0	0	0	6 ± 0.7	*Staphylococcus aureus*
-	29 ± 2.8	-	-	-	-	-	0	0	0	16 ± 2.8	0	0	0	0	10 ± 1.4	*Klebsiella pneumoniae*
-	23 ± 0.7	-	-	10 ± 0.7	-	-	0	0	0	0	7 ± 1.4	7 ± 0.7	0	0	9 ± 2.8	*Acinetobacter baumanii*
19 ± 1.4	-	-	-	-	-	-	0	0	0	15 ± 0.7	14 ± 2.8	16 ± 0.7	13 ± 1.4	15 ± 0.7	16 ± 0.7	*Candida tropicalis*
16 ± 0.7	-	-	-	-	-	-	8 ± 0.7	0	0	17 ± 2.8	0	0	0	12 ± 1.4	15 ± 1.4	*Candida albicans*
19 ± 1.4	-	-	-	-	-	-	0	0	0	12 ± 0.7	10 ± 2.8	10 ± 0.7	11 ± 1.4	11 ± 0.7	12 ± 0.7	*Candida glabrata*

## Supplementary Materials

The following are available online at http://www.mdpi.com/1420-3049/23/4/851/s1, Figure S1: ^1^H-NMR spectrum of compound **1** in CDCl_3_, Figure S2: ^13^C-Jmod-NMR spectrum of compound **1** in CDCl_3_, Figure S3: ^1^H-^1^H COSY spectrum of compound **1** in CDCl_3_, Figure S4: ^1^H-^13^C HSQC spectrum of compound **1** in CDCl_3_, Figure S5: ^1^H-NMR spectrum of compound **2** in CDCl_3_, Figure S6: ^13^C-Jmod-NMR spectrum of compound **2** in CDCl_3_, Figure S7: ^1^H-NMR spectrum of compound **3** in DMSO, Figure S8: ^13^C-Jmod-NMR spectrum of compound **3** in DMSO, Figure S9: ^1^H-^13^C HMBC spectrum of compound **3** in DMSO, Figure S10: ^1^H-NMR spectrum of compound **4** in CD_3_OD, Figure S11: ^1^H-^1^H COSY spectrum of compound **4** in CD_3_OD, Figure S12: ^1^H-^13^C HSQC spectrum of compound **4** in CD_3_OD, Figure S13: ^1^H-^1^H COSY spectrum of compound **5** in CD_3_OD, Figure S14: ^13^C-Jmod-NMR spectrum of compound **5** in CD_3_OD, Figure S15: ^1^H-^13^C HSQC spectrum of compound **5** in CD_3_OD, Figure S16: ^1^H-^13^C HMBC spectrum of compound **5** in CD_3_OD.

## References

[1] Lattanzi E. (2008). The distribution of three species of the genus *Calicotome* in Italy. Flora Mediterr..

[2] Loy G., Cottiglia F., Garau D., Deidda D., Pompei R., Bonsignore L. (2001). Chemical composition and cytotoxic and antimicrobial activity of *Calycotome villosa* (Poiret) link leaves. Il Farmaco.

[3] Antri A.E., Messouri I., Tlemçani R.C., Bouktaib M., El Alami R., El Bali B., Lachkar M. (2004). Flavone glycosides from *Calycotome villosa* subsp. Intermedia. Molecules.

[4] Elkhamlichi A., El Antri A., El Hajaji H., El Bali B., Oulyadi H., Lachkar M. (2014). Phytochemical constituents from the seeds of *Calycotome villosa* subsp. Intermedia. Arab. J. Chem..

[5] Tohmé G., Tohmé H., Hamzé M. (2007). Illustrated Flora of Lebanon.

[6] Said O., Khalil K., Fulder S., Azaizeh H. (2002). Ethnopharmacological survey of medicinal herbs in israel, the golan heights and the west bank region. J. Ethnopharmacol..

[7] Pistelli L., Fiumi C., Morelli I., Giachi I. (2003). Flavonoids from *Calicotome villosa*. Fitoterapia.

[8] Wansi J.D., Bavoua D., Liliane J., Happi E.N., Devkota K.P., Lenta B.N., Mesaik M.A., Choudhary M.I., Fomum Z.T., Sewald N. (2009). Bioactive phenylethanoids and coumarines from *Basalmocitrus cameroonensis*. Z. Naturforsch. B.

[9] Kamboj A., Saluja A.K. (2011). Isolation of stigmasterol and β-sitosterol from petroleum ether extract of aerial parts of *Ageratum conyzoides* (asteraceae). Int. J. Pharm. Pharm. Sci..

[10] Jamal A., Yaacob W., Din L.B. (2009). A chemical study on *Phyllanthus columnaris*. Eur. J. Sci. Res..

[11] Pierre L.L., Moses M.N. (2015). Isolation and characterisation of stigmasterol and β-sitosterol from *Odontonema strictum* (acanthaceae). J. Innov. Pharm. Biol. Sci..

[12] Govindarajan P., Sarada D. (2011). Isolation and characterization of stigmasterol and β-sitosterol from *Acacia nilotica* (L.) *delile* ssp *indica* (benth.) brenan. J. Pharm. Res..

[13] Habib M., Nikkon F., Rahman M., Haque Z., Karim M. (2007). Isolation of stigmasterol and β-sitosterol from methanolic extract of root bark of *Calotropis gigantea*. Pak. J. Biol. Sci..

[14] Liu H., Mou Y., Zhao J., Wang J., Zhou L., Wang M., Wang D., Han J., Yu Z., Yang F. (2010). Flavonoids from *Halostachys caspica* and their antimicrobial and antioxidant activities. Molecules.

[15] Liu Q., Dixon R.A., Mabry T.J. (1993). Additional flavonoids from elicitor-treated cell cultures of *Cephalocereus senilis*. Phytochemistry.

[16] De Rosa S., De Stefano S. (1983). Chrysin 7-gentiobioside from the flowers of *Spartium junceum*. Phytochemistry.

[17] Beutler J.A., Cardellina J.H., Gray G.N., Prather T.R., Shoemaker R.H., Boyd M.R., Lin C.M., Hamel E., Cragg G.M. (1993). Two new cytotoxic chalcones from *Calythropsis aurea*. J. Nat. Prod..

[18] Amri B., Martino E., Vitulo F., Corana F., Kaâb L.B.-B., Rui M., Rossi D., Mori M., Rossi S., Collina S. (2017). *Marrubium*
*vulgare* L. Leave extract: Phytochemical composition, antioxidant and wound healing properties. Molecules.

[19] Sreeramulu D., Reddy C.V.K., Chauhan A., Balakrishna N., Raghunath M. (2013). Natural antioxidant activity of commonly consumed plant foods in India: Effect of domestic processing. Oxid. Med. Cell. Longev..

[20] Graham S.W., Reeves P.A., Burns A.C., Olmstead R.G. (2000). Microstructural changes in noncoding chloroplast DNA: Interpretation, evolution, and utility of indels and inversions in basal angiosperm phylogenetic inference. Int. J. Plant Sci..

[21] Al-Hadhrami R.M.S., Hossain M.A. (2016). Evaluation of antioxidant, antimicrobial and cytotoxic activities of seed crude extracts of *Ammi majus* grown in oman. Egypt. J. Basic Appl. Sci..

[22] Dessi M.A., Deiana M., Rosa A., Piredda M., Cottiglia F., Bonsignore L., Deidda D., Pompei R., Corongiu F.P. (2001). Antioxidant activity of extracts from plants growing in sardinia. Phytother. Res. PTR.

[23] Ali R.M., Houghton P.J. (1999). A new phenolic fatty acid ester with lipoxygenase inhibitory activity from *Jacaranda filicifolia*. Planta Med..

[24] Wei X., Yang C., Liang J. (2005). Constituents of the barks of *Fraxinus chinensis* roxb. Chin. J. Nat. Med..

[25] Eyong K.O., Folefoc G.N., Kuete V., Beng V.P., Krohn K., Hussain H., Nkengfack A.E., Saeftel M., Sarite S.R., Hoerauf A. (2006). Newbouldiaquinone a: A naphthoquinone–anthraquinone ether coupled pigment, as a potential antimicrobial and antimalarial agent from *Newbouldia laevis*. Phytochemistry.

[26] Kuete V., Eyong K., Folefoc G., Beng V., Hussain H., Krohn K., Nkengfack A. (2007). Antimicrobial activity of the methanolic extract and of the chemical constituents isolated from newbouldia laevis. Die Pharm. Int. J. Pharm. Sci..

[27] Eyong K.O., Krohn K., Hussain H., Folefoc G.N., Nkengfack A.E., Schulz B., Hu Q. (2005). Newbouldiaquinone and newbouldiamide: A new naphthoquinone–anthraquinone coupled pigment and a new ceramide from *Newbouldia laevis*. Chem. Pharm. Bull..

[28] Vouffo B., Hussain H., Eyong K.O., Dongo E., Folefoc G.N., Nkengfack A.E., Krohn K. (2008). Chemical constituents of *Dorstenia picta* and *Newbouldia laevis*. Biochem. Syst. Ecol..

[29] Zhenxi Y., Gangli W., Zhong D., Ciren B., Ruichao L. (2007). Chemical constituents of *Phlomis medicinalis* ii. Zhongguo Yaoxue Zazhi (Beijing, China).

[30] Pereira V.V., Silva R.R., Duarte L.P., Takahashi J.A. (2016). Chemical constituents of *Jacaranda oxyphylla* and their acetylcholinesterase inhibitory and antimicrobial activities. Rec. Nat. Prod..

[31] Jamaluddin F., Mohamed S., Lajis M.N. (1994). Hypoglycaemic effect of *Parkia speciosa* seeds due to the synergistic action of β-sitosterol and stigmasterol. Food Chem..

[32] Jain S.C., Singh B., Jain R. (2001). Antimicrobial activity of triterpenoids from *Heliotropium ellipticum*. Fitoterapia.

[33] Singh B., Dubey M.M. (2001). Estimation of triterpenoids from *Heliotropium marifolium* koen. Ex retz. In vivo and in vitro. I. Antimicrobial screening. Phytother. Res..

[34] Suhaj M. (2006). Spice antioxidants isolation and their antiradical activity: A review. J. Food Compos. Anal..

[35] Kaur N., Chaudhary J., Jain A., Kishore L. (2011). Stigmasterol: A comprehensive review. Int. J. Pharm. Sci. Res..

[36] Saeidnia S., Manayi A., Gohari A.R., Abdollahi M. (2014). The story of beta-sitosterol—A review. Eur. J. Med. Plants.

[37] Pearl I.A., Darling S.F. (1971). Barks of the family salicaceae. Xxvi. Hot water extractives of the bark and leaves of populus deltoides. Can. J. Chem..

[38] Glyzin V.I., Ban’kovskii A.I., Pakaln D.A. (1975). Flavonoids of *Scutellaria orientalis* and *Scutellaria karjagini*. Chem. Nat. Compd..

[39] Ogawa M., Ogihara Y. (1976). Constituents of *Enkianthus subsessilis* (miquel) makino subspecies nudipes (honda) kitamura. 6. Constituents of the leaves, examination of koaburanin and relation with constituents and taxonomy of *E. Subsessilis* subspecies *subsessilis*, an intraspecific variety of *E. subsessilis* subspecies *nudipes*. Shoyakugaku Zasshi.

[40] Brum-Bousquet M., Tillequin F., Paris R.R. (1976). Flavonic pigments of *Sarothamnus patens*. Presence of chrysin 7-β-monoglucoside. Planta Med..

[41] Gellert M., Szendrei K., Dinya Z., Repasi J. (1986). Characteristic constituents of the cherry peduncle extract novicardin: Hplc fingerprint. Stud. Org. Chem. (Amst.).

[42] Cherkaoui-Tangi K., Lachkar M., Wibo M., Morel N., Gilani A.H., Lyoussi B. (2008). Pharmacological studies on hypotensive, diuretic and vasodilator activities of chrysin glucoside from *Calycotome villosa* in rats. Phytother. Res..

[43] Berthier A., Girard C., Grandvuillemin A., Muyard F., Skaltsounis A.-L., Jouvenot M., Delage-Mourroux R. (2007). Effect of 7-*O*-β-d-glucopyranosylchrysin and its aglycone chrysin isolated from *Podocytisus caramanicus* on estrogen receptor α transcriptional activity. Planta Med..

[44] Ndjateu F.S.T., Tsafack R.B.N., Nganou B.K., Awouafack M.D., Wabo H.K., Tene M., Tane P., Eloff J.N. (2014). Antimicrobial and antioxidant activities of extracts and ten compounds from three cameroonian medicinal plants: *Dissotis perkinsiae* (melastomaceae), *Adenocarpus mannii* (fabaceae) and *Barteria fistulosa* (passifloraceae). S. Afr. J. Bot..

[45] Oki T., Matsui T., Osajima Y. (1999). Inhibitory effect of α-glucosidase inhibitors varies according to its origin. J. Agric. Food Chem..

[46] Önal S., Timur S., Okutucu B., Zihnioğlu F. (2005). Inhibition of α-glucosidase by aqueous extracts of some potent antidiabetic medicinal herbs. Prep. Biochem. Biotechnol..

[47] Wang W., Zhao Y., Rayburn E.R., Hill D.L., Wang H., Zhang R. (2007). In vitro anti-cancer activity and structure–activity relationships of natural products isolated from fruits of panax ginseng. Cancer Chemother. Pharmacol..

[48] Chen Y., Wang H., Xu S. (1995). Study on the chemical constituents of panax ginseng and their structure–function relationship anti-arrythmia and anti-tumor. Sci. Found. China.

[49] Ross S.A., Zagloul A., Nimrod A.C., Mehmedic Z., El Sohly H.N. (1999). A cytotoxic chalcone from *Faramea salicifolia*. Planta Med..

[50] Wu Z., Wang B., Zhao Y., Yang X., Liang H. (2009). Chalcones from *Bauhinia glauca* subsp. *Pernervosa*. China J. Chin. Mater. Med..

[51] Sogawa S., Nihro Y., Ueda H., Izumi A., Miki T., Matsumoto H., Satoh T. (1993). 3,4-dihydroxychalcones as potent 5-lipoxygenase and cyclooxygenase inhibitors. J. Med. Chem..

[52] Seguin E., Elomri A., Magiatis P., Skaltsounis A.-L., Chao L.R., Tillequin F. (2002). Synthesis, dimerization, and biological activity of hexaoxygenated chalcones related to calythropsin and combretastatins. Nat. Prod. Lett..

[53] Rullah K., Aluwi M.F.F.M., Yamin B.M., Bahari M.N.A., Wei L.S., Ahmad S., Abas F., Ismail N.H., Jantan I., Wai L.K. (2014). Inhibition of prostaglandin e 2 production by synthetic minor prenylated chalcones and flavonoids: Synthesis, biological activity, crystal structure, and in silico evaluation. Bioorg. Med. Chem. Lett..

[54] Kaufmann K.B., Ulbrich F., Schallner N., Goebel U., Al-Rifai N., Rucker H., Enzinger M., Petkes H., Pitzl S., Amslinger S. (2015). The cytoprotective effects of e-α-(4-methoxyphenyl)-2′,3,4,4′-tetramethoxychalcone (e-α-p-ome-c6h4-tmc)—A novel and non-cytotoxic ho-1 inducer. PLoS ONE.

[55] Ruecker H., Amslinger S. (2015). Identification of heme oxygenase-1 stimulators by a convenient elisa-based bilirubin quantification assay. Free Radic. Biol. Med..

[56] Morel I., Lescoat G., Cogrel P., Sergent O., Pasdeloup N., Brissot P., Cillard P., Cillard J. (1993). Antioxidant and iron-chelating activities of the flavonoids catechin, quercetin and diosmetin on iron-loaded rat hepatocyte cultures. Biochem. Pharmacol..

[57] Sahu N.K., Balbhadra S.S., Choudhary J., Kohli D.V. (2012). Exploring pharmacological significance of chalcone scaffold: A review. Curr. Med. Chem..

[58] Chandini S.K., Ganesan P., Bhaskar N. (2008). In vitro antioxidant activities of three selected brown seaweeds of India. Food Chem..

[59] Yen G.-C., Chen H.-Y. (1995). Antioxidant activity of various tea extracts in relation to their antimutagenicity. J. Agric. Food Chem..

[60] Tereschuk M.A.L., Riera M.V., Castro G.R., Abdala L.R. (1997). Antimicrobial activity of flavonoids from leaves of *Tagetes minuta*. J. Ethnopharmacol..

[61] Accary C., Rima M., Kouzayha A., Hleihel W., Sadek R., Desfontis J.C., Fajloun Z., Hraoui-Bloquet S. (2014). Effect of the *Montivipera bornmuelleri* snake venom on human blood: Coagulation disorders and hemolytic activities. Open J. Hematol..

[62] Kumar D., Kumar H., Vedasiromoni J.R., Pal B.C. (2012). Bio-assay guided isolation of α-glucosidase inhibitory constituents from *Hibiscus mutabilis* leaves. Phytochem. Anal..

[63] de Araújo A.L., Radvanyi F. (1987). Determination of phospholipase A2 activity by a colorimetric assay using a ph indicator. Toxicon.

[64] Strober W. (2001). Trypan blue exclusion test of cell viability. Curr. Protoc. Immunol..

