# Supplementary material for: Isolation of Bioactive Compounds from Calicotome villosa Stems
Source: Molecules. 2018 Apr 8;23(4):851. doi: 10.3390/molecules23040851 (PMC6017485; doi:10.3390/molecules23040851)
Supplement: Supplementary file 1 [file molecules-23-00851-s001.pdf]

Supplementary data

Isolation of Bioactive compounds from *Calicotome villosa* Stems

Josiane Alhage [1,2], Hoda Elbitar [1,*], Samir Taha [1,3], Jean-Paul Guegan [2], Zeina Dassouki [1], Thomas Vives [2] and Thierry Benvegnu [2,*]

[1] AZM Centre for Research in Biotechnology and Its Applications, Laboratory of Applied Biotechnology for Biomolecules, Biotherapy and Bioprocess, Lebanese University, El Mitein Street, Tripoli, Lebanon; josiane.alhage@ensc-rennes.fr (J.A.); samirtaha@ul.edu.lb (S.T.); zeina_dassouki@hotmail.com (Z.D.)

[2] ENSCR, CNRS, ISCR (Institut des Sciences Chimiques de Rennes)–UMR 6226, Univ Rennes, 35000 Rennes, France; jean-paul.guegan@ensc-rennes.fr (J.-P.G.); thomas.vives@ensc-rennes.fr (T.V.)

[3] Faculty of Public Health, Rafic Hariri Campus, Lebanese University, Hadath, Beyrouth, Lebanon

* Correspondence: elbitarhoda@yahoo.fr (H.E.); thierry.benvegnu@ensc-rennes.fr (T.B.); Tel.: +961-3-522-303 (H.E.); +33-223-238-060 (T.B.);

Contents

Supplementary Figures

Figure S1: $^{\text{1H-NMR}}$ spectrum of compound **1** in CDCl3

Figure S2: $^{\text{13C-Jmod}}$ - NMR spectrum of compound **1** in CDCl3

Figure S3: $^{\text{1H-1H}}$ COSY spectrum of compound **1** in CDCl3

Figure S4: $^{\text{1H-13C}}$ HSQC spectrum of compound **1** in CDCl3

Figure S5: $^{\text{1H-NMR}}$ spectrum of compound **2** in CDCl3

Figure S6: $^{\text{13C-Jmod}}$ - NMR spectrum of compound **2** in CDCl3

Figure S7: $^{\text{1H-NMR}}$ spectrum of compound **3** in DMSO

Figure S8: $^{\text{13C-Jmod}}$ - NMR spectrum of compound **3** in DMSO

Figure S9: $^{\text{1H-13C}}$ HMBC spectrum of compound **3** in DMSO

Figure S10: $^{\text{1H-NMR}}$ spectrum of compound **4** in CD3OD

Figure S11: $^{\text{1H-1H}}$ COSY spectrum of compound **4** in CD3OD

Figure S12: $^{\text{1H-13C}}$ HSQC spectrum of compound **4** in CD3OD

Figure S13: $^{\text{1H-1H}}$ COSY spectrum of compound **5** in CD3OD

Figure S14: $^{\text{13C-Jmod}}$ - NMR spectrum of compound **5** in CD3OD

Figure S15: $^{\text{1H-13C}}$ HSQC spectrum of compound **5** in CD3OD

Figure S16: $^{\text{1H-13C}}$ HMBC spectrum of compound **5** in CD3OD

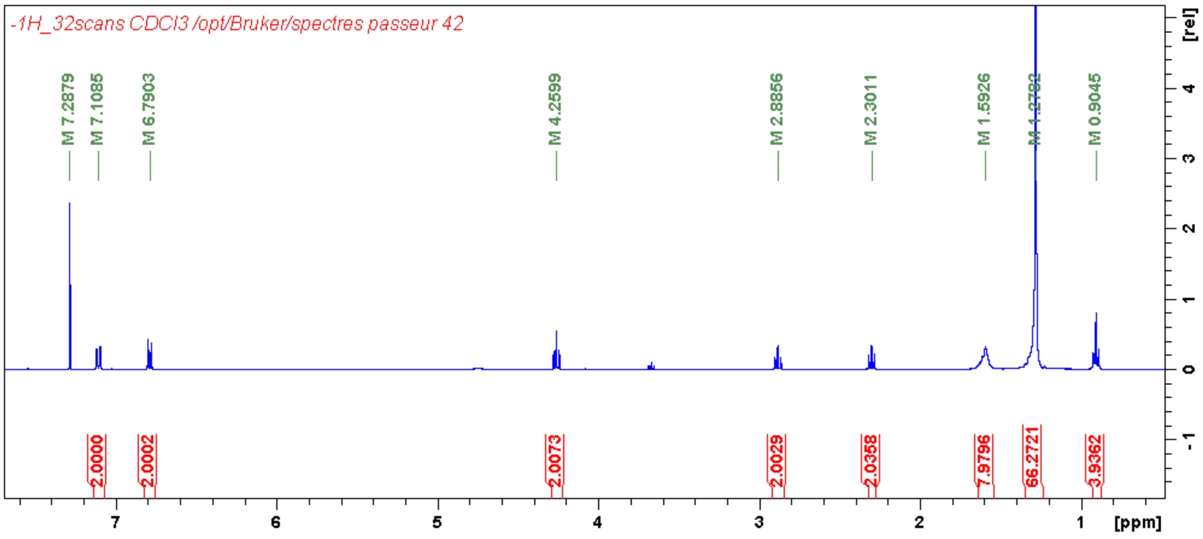

Figure S1: ^{1}H-NMR spectrum of compound **1** in $CDCl_3$, 25°C at 400.13 MHz

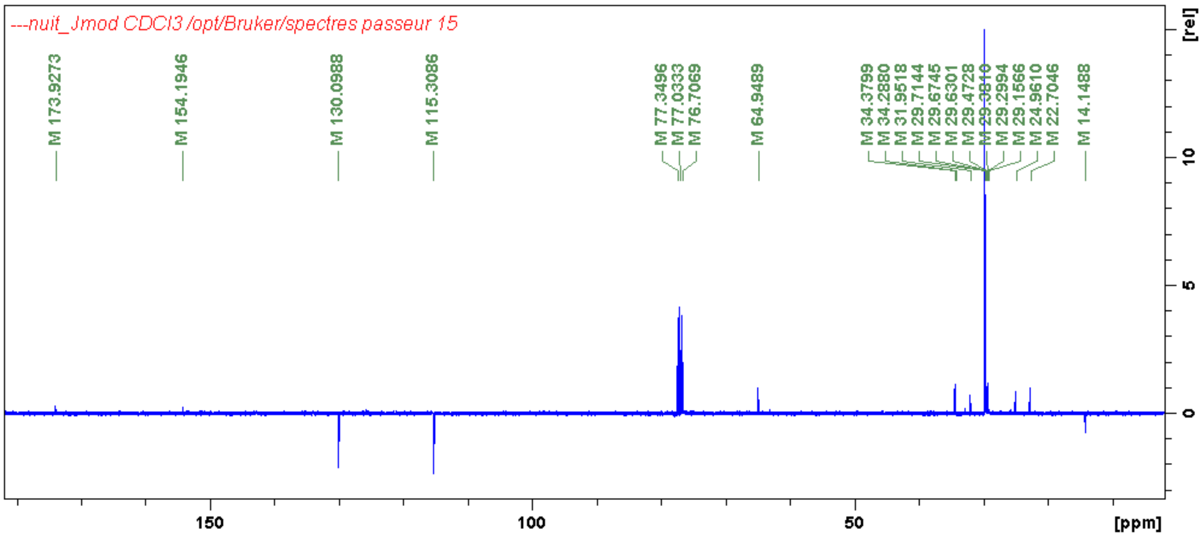

Figure S2: ^{13}C-Jmod - NMR spectrum of compound **1** in $CDCl_3$, 25°C at 100.13 MHz

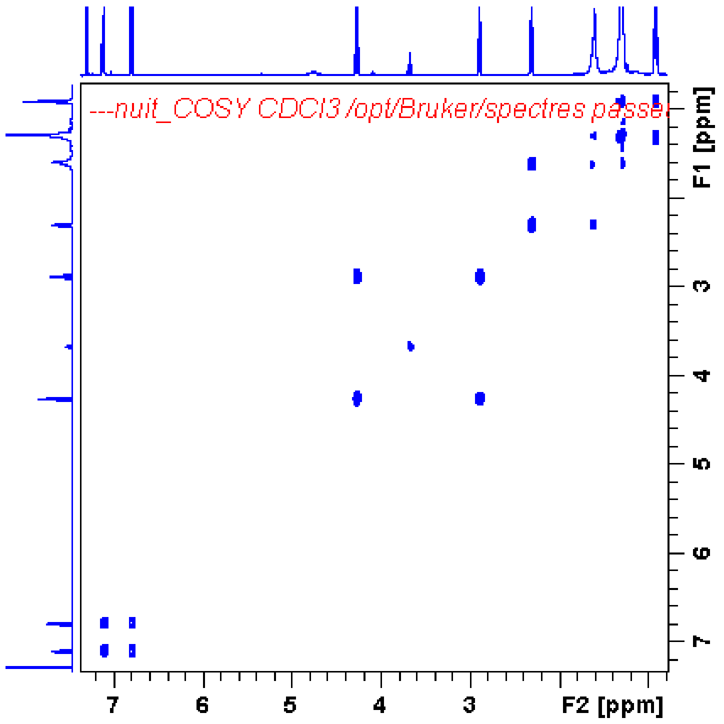

Figure S3: ^{1}H-^{1}H COSY spectrum of compound **1** in $CDCl_3$, 25°C at 400.13 MHz

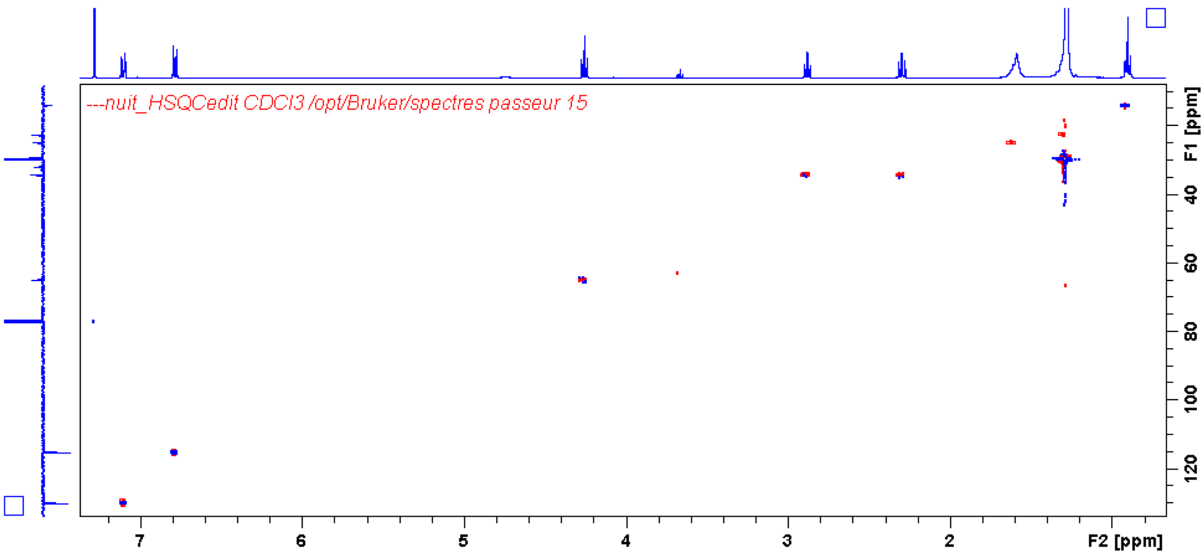

Figure S4: ^{1}H-^{13}C HSQC spectrum of compound **1** in $CDCl_3$, 25°C at 400.13 MHz for ^{1}H

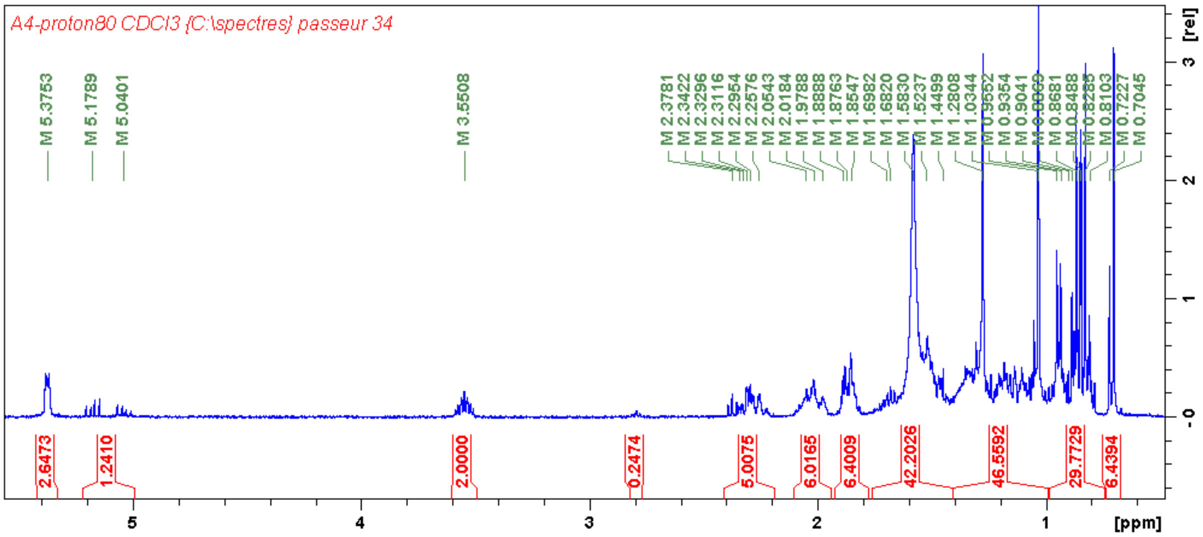

Figure S5: ^{1}H-NMR spectrum of compound **2** in $CDCl_3$, 25°C at 400.13 MHz

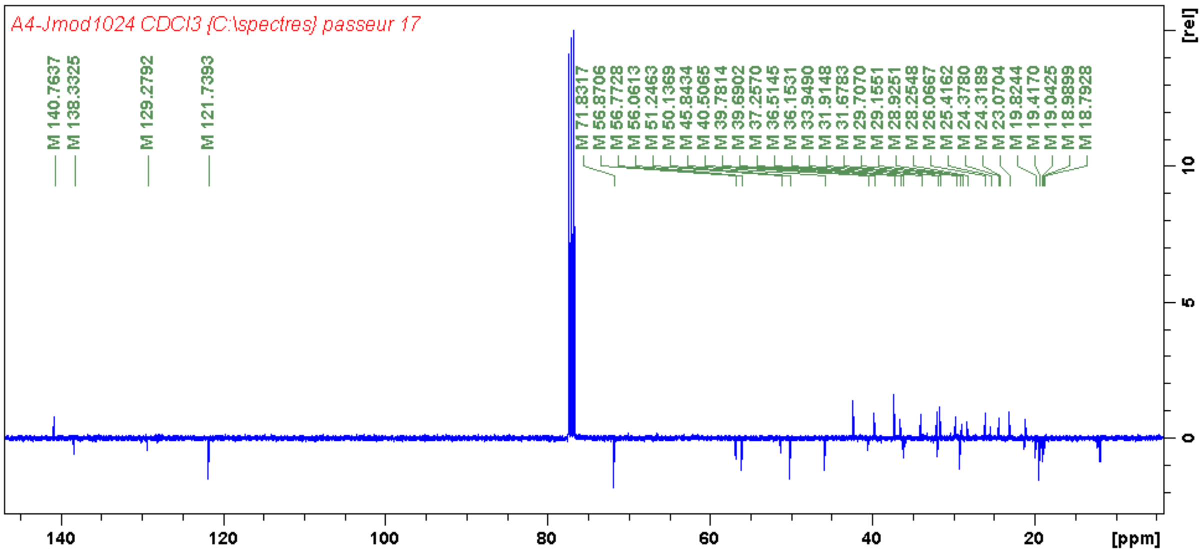

Figure S6: ^{13}C-Jmod - NMR spectrum of compound **2** in $CDCl_3$, 25°C at 100.13 MHz

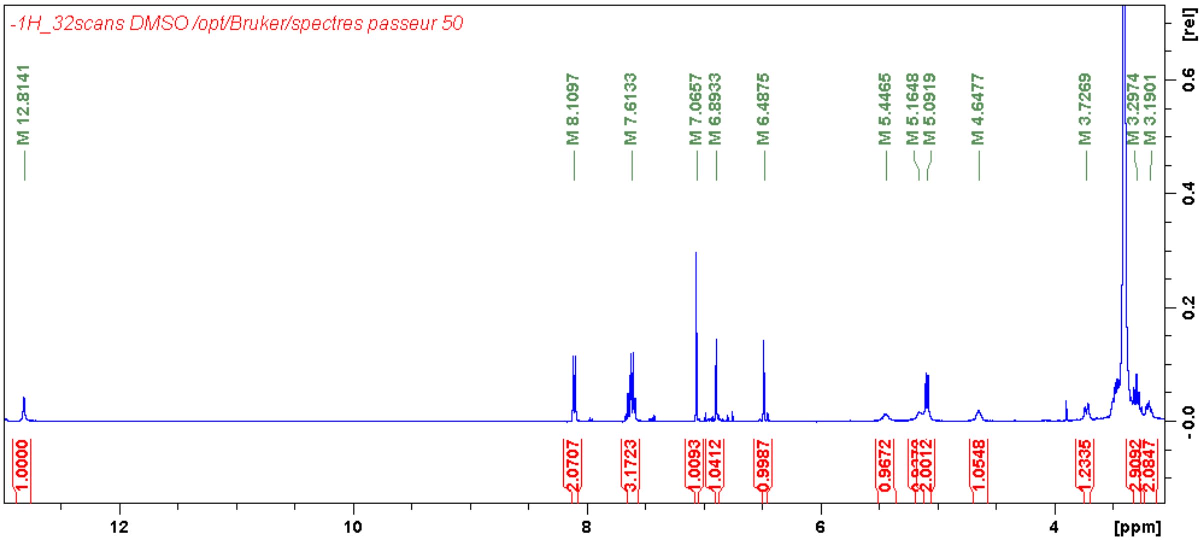

Figure S7: ^{1}H-NMR spectrum of compound **3** in DMSO, 25°C at 400.13 MHz

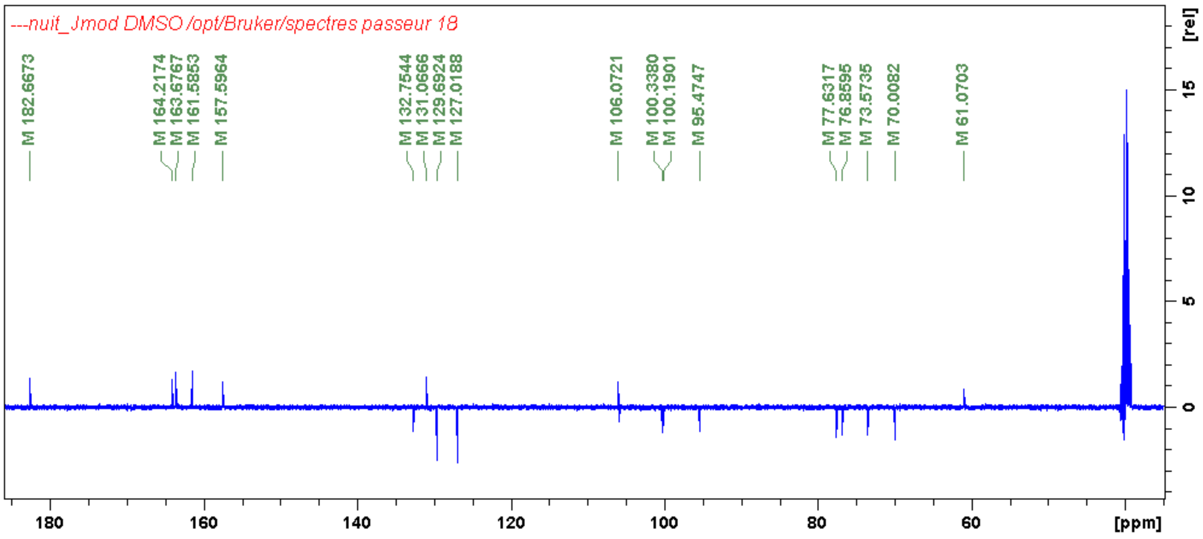

Figure S8: ^{13}C-Jmod - NMR spectrum of compound **3** in DMSO, 25°C at 100.13 MHz

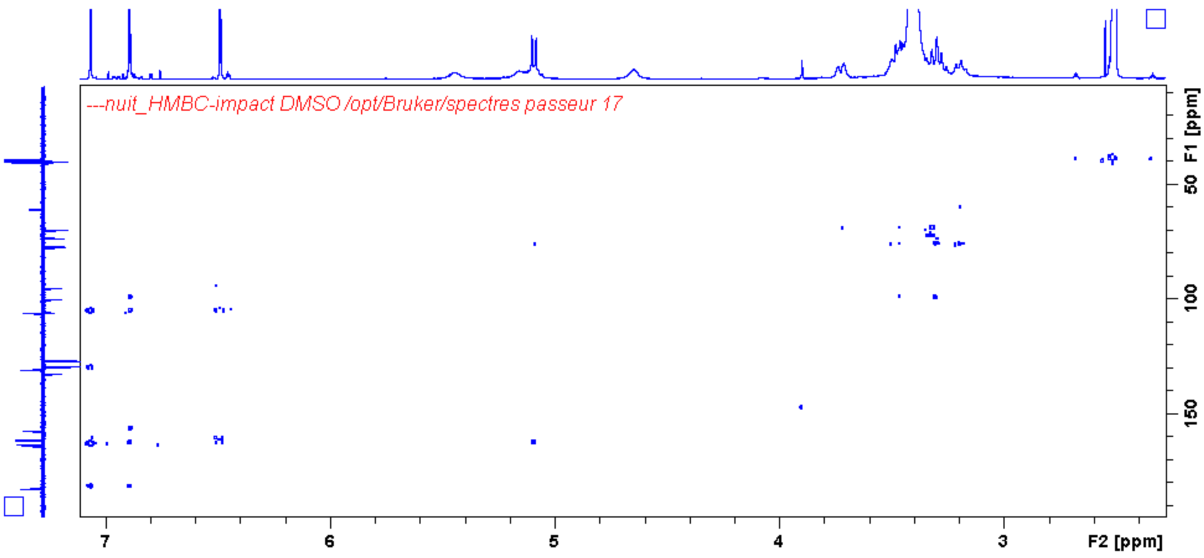

Figure S9: ^{1}H-^{13}C HMBC spectrum of compound **3** in DMSO, 25°C at 400.13 MHz for ^{1}H

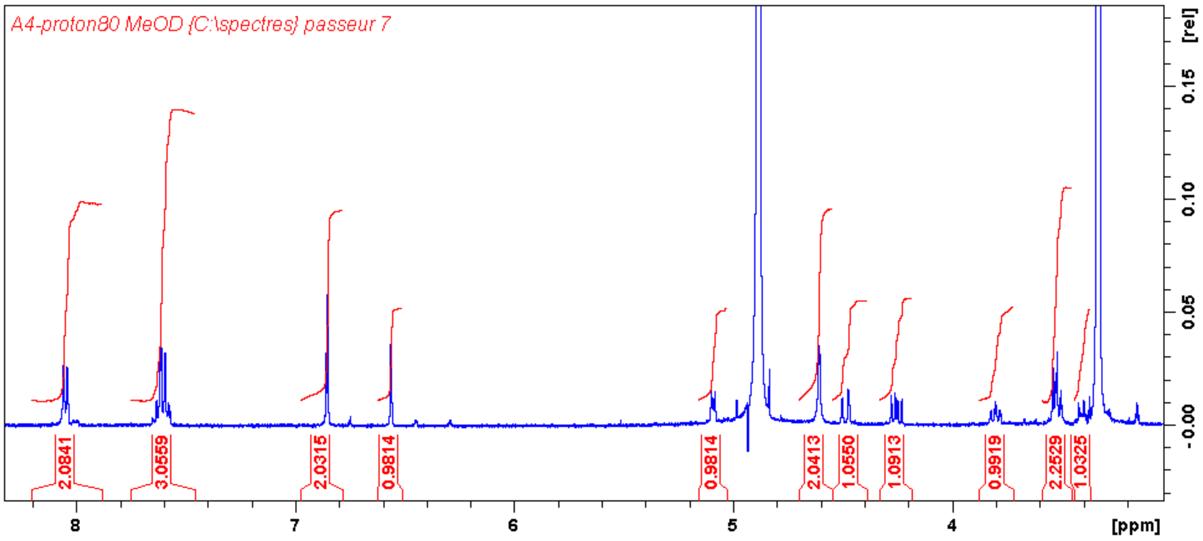

Figure S10: ^{1}H-NMR spectrum of compound **4** in CD_3OD, 25°C at 400.13 MHz

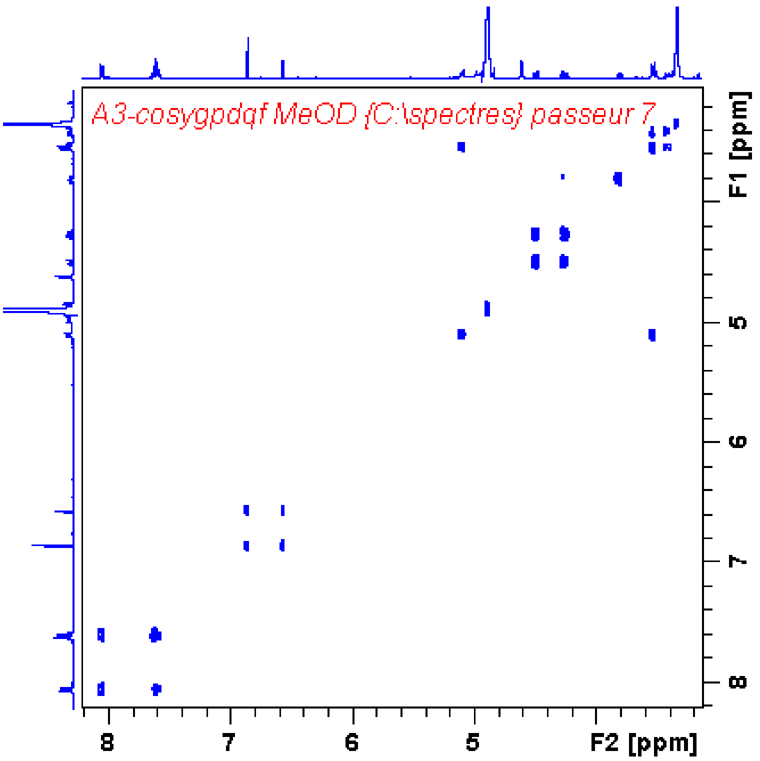

Figure S11: ^{1}H-^{1}H COSY spectrum of compound **4** in CD_3OD, 25°C at 400.13 MHz

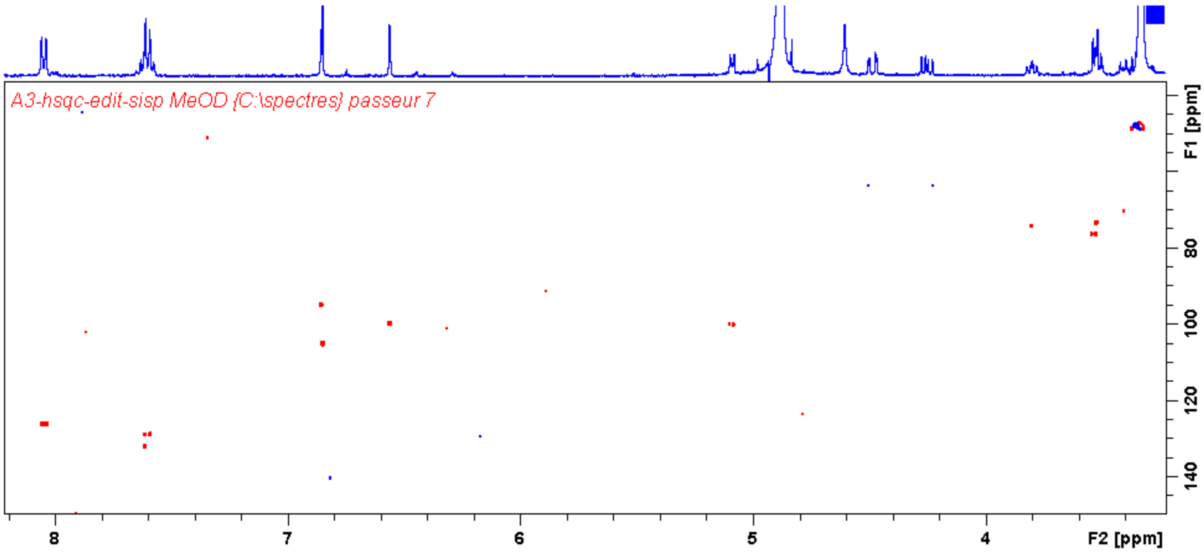

Figure S12: ^{1}H-^{13}C HSQC spectrum of compound **4** in CD_3OD, 25°C at 400.13 MHz for ^{1}H

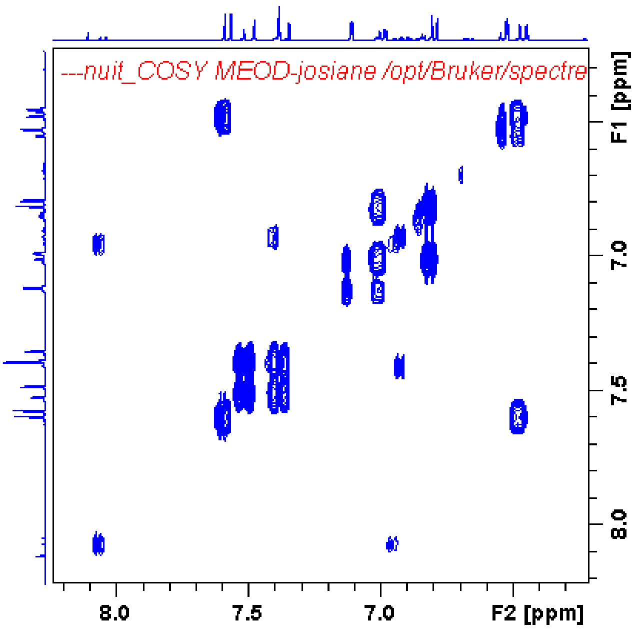

Figure S13: ^1H-^1H COSY spectrum of compound **5** in CD_3OD, 25°C at 400.13 MHz

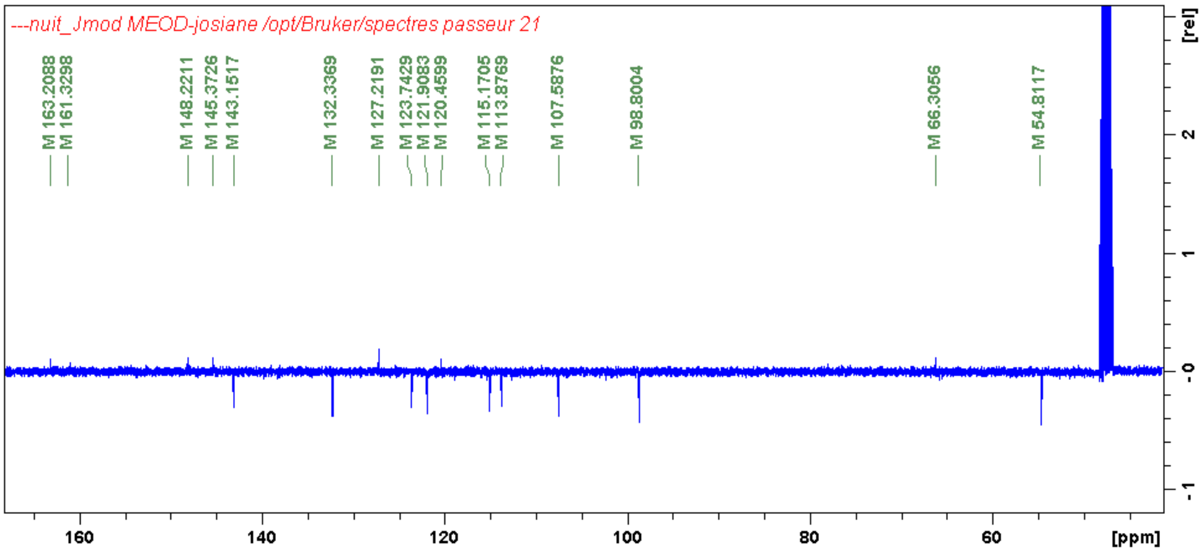

Figure S14: ^{13}C-Jmod - NMR spectrum of compound **5** in CD_3OD, 25°C at 100.13 MHz

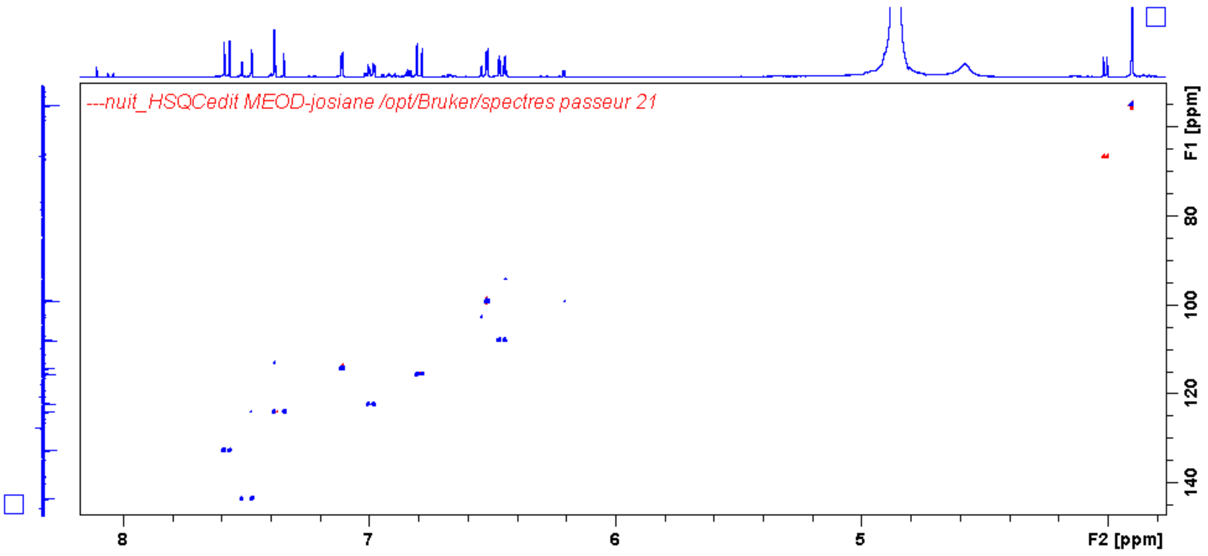

Figure S15: ^{1}H-^{13}C HSQC spectrum of compound **5** in CD_3OD, 25°C at 400.13 MHz for ^{1}H

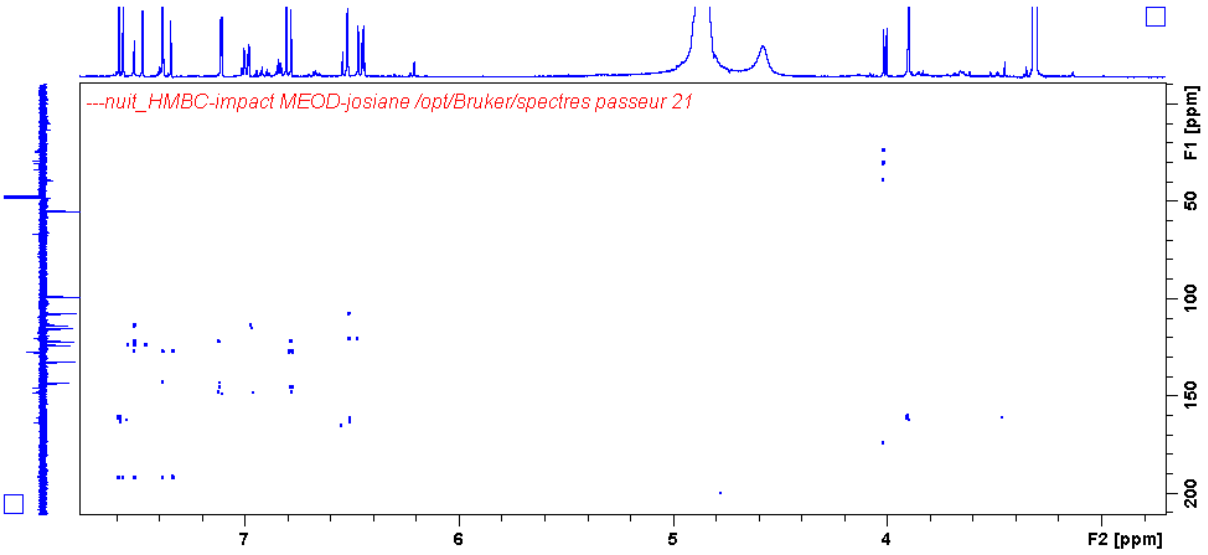

Figure S16: ^{1}H-^{13}C HMBC spectrum of compound **5** in CD_3OD, 25°C at 400.13 MHz for ^{1}H
